# The role of endothelial cells in pancreatic islet development, transplantation and culture

**DOI:** 10.3389/fcell.2025.1558137

**Published:** 2025-04-22

**Authors:** Ajun Geng, Shubo Yuan, Qing Cissy Yu, Yi Arial Zeng

**Affiliations:** ^1^ Key Laboratory of Systems Health Science of Zhejiang Province, School of Life Science, Hangzhou Institute for Advanced Study, University of Chinese Academy of Sciences, Hangzhou, China; ^2^ New Cornerstone Science Laboratory, Key Laboratory of Multi-Cell Systems, Shanghai Institute of Biochemistry and Cell Biology, Center for Excellence in Molecular Cell Science, University of Chinese Academy of Sciences, Chinese Academy of Sciences, Shanghai, China

**Keywords:** endothelial cell (EC), pancreatic islet, diabetes mellitus, organoid culture, islet transplantation, β cells

## Abstract

Endothelial cells (ECs) play pivotal roles in the development and maintenance of tissue homeostasis. During development, vasculature actively involves in organ morphogenesis and functional maturation, through the secretion of angiocrine factors and extracellular matrix components. Islets of Langerhans, essential functional units of glucose homeostasis, are embedded in a dense endothelial capillary network. Islet vasculature not only supplies nutrients and oxygen to endocrine cells but also facilitate the rapid delivery of pancreatic hormones to target tissues, thereby ensuring precise glucose regulation. Diabetes mellitus is a major disease burden and is caused by islet dysfunction or depletion, often accompanied by vessel loss and dysregulation. Therefore, elucidating the regulatory mechanisms of ECs within islets hold profound implications for diabetes therapy. This review provides an overview of recent research advancements on the functional roles of ECs in islet biology, transplantation, and *in vitro* islet organoid culture.

## 1 Introduction

Pancreatic islets, also known as islets of Langerhans, are the primary functional unit for blood glucose regulation, through the finetuning of various secreted hormones. Islets consist of four main endocrine cell types: β cells, α cells, δ cells, and PP cells, which secrete insulin, glucagon, somatostatin, and pancreatic polypeptide, respectively (Nadal et al., 1999). Islets are embedded in dense capillary networks, which are established alongside embryonic islet formation and actively maintain islet function (Cleaver and Dor, 2012; Burganova et al., 2021; Hogan and Hull, 2017). Despite constituting only 1%–2% of pancreatic tissue volume, islets disproportionately receive 10%–15% of the pancreatic blood flow (Henderson and Moss, 1985), which highlights the critical function of islet vasculature in warranting efficient nutrient delivery, precise glucose sensing, and rapid hormone distribution.

Diabetes mellitus, characterized by insufficient insulin secretion or impaired insulin response, results in uncontrollable hyperglycemia. Conventional treatments, such as glucagon-like peptide-1 (GLP-1) receptor agonists and insulin injections, require burdensome daily long-term intervention. Therefore, islet transplantation has emerged as a promising approach for restoring autonomous glycemic regulation. However, many challenges persist, including donor shortages, immune rejection, and the unpredictable survival of post-transplantation islets (Pepper et al., 2018; Shapiro et al., 2000; Lysy et al., 2013). ECs have been demonstrated to enhance islet survival post-transplantation, emphasizing the importance of vascularization in transplantation success (Bowers et al., 2019; Chen et al., 2023). In this review, we summarize recent studies explicating the role of ECs in islet biology, transplantation, and *in vitro* organoid culturing, combined with newly emerging technologies to further clinical translation for diabetes treatment.

## 2 ECs in pancreatic islet development

**FIGURE 1:**
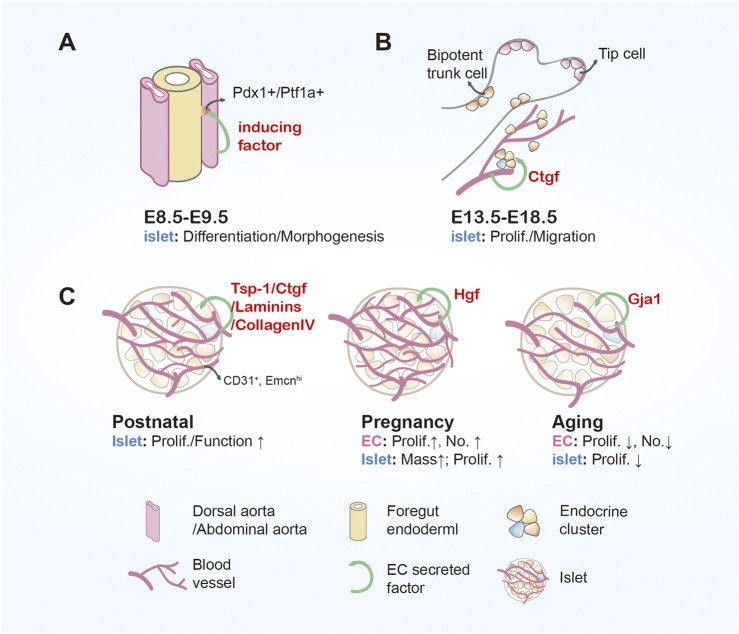
Endothelial cell in pancreatic islet development **(A)** During embryonic days 8.5–9.5, dorsal and ventral foregut epithelia show budding protrusions, forming dorsal and ventral pancreatic buds. Endothelial cells secrete factors that induce the formation of Pdx1/Ptf1a^+^ cells. **(B)** During embryonic development from days 13.5–18.5, the pancreas undergoes a secondary transition where endocrine cells are produced and differentiated, and subsequently separate from the ducts, migrating to form islets. Endothelial cells can secrete Ctgf to promote the proliferation and migration of β cells. **(C)** At postnatal stage, endothelial cells not only secrete cell factors (Tsp-1, Ctgf) that regulate islet cell development but also participate in the regulation of β-cell function, proliferation, and expansion through various proteins (laminins/collagen IV) in the vascular basement membrane. As adults, the pancreas undergoes gradual updates, and the capacity of pancreatic beta cells is subject to dynamic changes. With metabolic changes in the body, such as pregnancy, aging, endocrine cells and endothelial cells also undergo corresponding changes. Endothelial cell (EC); Pancreatic duodenal homeobox 1 (Pdx1); Pancreas associated transcription factor 1a (Ptf1a); Connective tissue growth factor (Ctgf); Thrombospondin-1 (Tsp-1); Hepatocyte growth factor (Hgf); Gap junction protein 1 (Gja1); Number (No.); Proliferation (Prolif.).

Signals derived from ECs are crucial for endocrine differentiation and islet functional maturation (Cleaver and Dor, 2012; Burganova et al., 2021; Hogan and Hull, 2017) (Figure 1). Here we enlist current studies illustrating the supporting role of ECs at various pancreatic developmental phases.

### 2.1 Role of ECs in islet embryonic development

As an endodermal origin, pancreas formation occurs alongside the major vessels (Cleaver and Dor, 2012). In mice, as early as embryonic day 8 (E8), direct contact was observed between dorsal aorta (red) and pancreatic foregut endoderm (yellow) (Figure 1A). Between E8.5 and E9.5, both dorsal and ventral pancreatic buds form and subsequently merge to generate the pancreas. Pancreatic epithelium underwent two distinct waves of endocrine differentiation. The initial wave, termed primary transition, begins around E9.5. During this phase, formed endocrine cells neither expressed mature markers nor assembled into functional islets. At around E13.5, secondary transition occurs, which marks a period of increased production and differentiation of endocrine cells with mature markers such as insulin by β cells and glucagon by α cells. By E18.5, these endocrine cells separate from ductal branches and migrate into the surrounding acinar tissue in aggregates to form mature islet structures (Pan and Wright, 2011; Gittes, 2009).

In humans, the dorsal and ventral pancreatic buds also arise from the foregut endoderm (Jennings et al., 2015), with their development initiating at 27–29 days post-conception (Jennings et al., 2013). Unlike in rodents, human pancreatic development involves only a single wave of endocrine cell formation. Furthermore, islet capillaries in humans are larger and less dense, with human islets containing five times fewer vessels per unit area compared to mouse islets (Brissova et al., 2015; Carlsson et al., 2002). A more comprehensive overview of ECs in human pancreas development can be found in this review (Henry et al., 2019). The mechanisms underlying the interaction between ECs and human islets remain unclear, as existing studies are primarily *in vitro* and lack *in vivo* validation.

During mouse embryonic development, ECs actively participated in pancreatic morphogenesis and endocrine differentiation (Figures 1A, B). In 2001, Lammer et al. showed that dorsal aorta induced simultaneous expression of pancreatic duodenal homeobox 1 (Pdx1), a key pancreatic transcription factor, and insulin, marker of β cells, in cultured endodermal cells (Lammert et al., 2001). Consistent with this, removal of the dorsal aorta in *Xenopus laevis* embryos significantly decreased or even completely abolished the expression of pro-endocrine transcription factors and prevented the emergence of insulin-expressing cells. Furthermore, transgenic mice with ectopic vascularization in the posterior foregut led to ectopic insulin expression and consequently islet hyperplasia, with the ectopically insulin-expressing cells closely associated with the vascularized areas (Lammert et al., 2001).

Interestingly, the critical role of ECs is more biased towards dorsal pancreatic bud development compared to the ventral part. Experiments using fetal liver kinase one knockout (*Flk1* ^*−/−*^) (Vascular endothelial growth factor receptor 2, *Vegfr2* ^*−/−*^) mouse embryos, which failed to form ECs, resulted in severe impairment of dorsal pancreatic budding and *Pdx1* expression, whereas ventral pancreatic development seemed unaffected (Yoshitomi and Zaret, 2004). Also, dorsal pancreatic epithelium in *Flk1* ^*−/−*^ embryos failed to activate pancreas-associated transcription factor 1a (Ptf1a) expression (Yoshitomi and Zaret, 2004), a key transcription factor for acquiring and maintaining pancreatic fate in progenitor cells (Kawaguchi et al., 2002).

In 2009, Crawford et al. discovered that connective tissue growth factor (Ctgf) is essential for establishing a normal ratio and architecture of islet endocrine cells during embryonic development (Crawford et al., 2009; Ivkovic et al., 2003). Specifically, *Ctgf* knockout mice displayed increased pancreatic Glucagon^+^ cells with concomitant decrease in Insulin^+^ cells, and disrupted separation of islets from the ductal epithelium (Crawford et al., 2009). Further study revealed that endothelial *Ctgf* deficiency led to reduced islet vascularization and decreased embryonic β cell proliferation (Guney et al., 2011) (Figure 1B).

ECs can also promote pancreatic development indirectly. Jacquemin et al. demonstrated that aortic ECs enhance the survival of mesenchymal cells surrounding the dorsal Islet1^+^ (a LIM-homeodomain transcription factor) cells, which secrete fibroblast growth factor 10 to promote *Ptf1a* expression in the pancreatic dorsal bud (Jacquemin et al., 2006). Kume and colleagues found that in chicken embryos, pancreatic endoderm cells secrete the C-X-C motif chemokine ligand 12, which recruits C-X-C chemokine receptor type 4-expressing angioblasts to migrate to the endodermal boundary, thereby inducing Pdx1 expression within the endoderm (Katsumoto and Kume, 2011). Overall, these studies elucidated ECs as an indispensable player in embryonic pancreas organogenesis.

### 2.2 Role of ECs in postnatal islet development

In 2006, Johansson et al. observed that the pronounced growth of islet endocrine cells during the first week after birth coincides with an even more significant increase in the proliferation of ECs, resulting in a marked increase in intra-islet vascular density. Furthermore, the proliferating endocrine cells were located near the islet ECs, suggesting a supportive role of islet vasculature in this phase of islet growth (Johansson et al., 2006a) (Figure 1C).

ECs have been shown to regulate islet β cell function through secreted factors including Thrombospondin-1 (Tsp-1) and Ctgf. In 2011, Johan Olerud and colleagues investigated the role of Tsp-1 in islet morphology and β cell function (Olerud et al., 2011). Tsp-1, a glycoprotein primarily produced by islet ECs, is a potent inhibitor of angiogenesis (Jimenez et al., 2000; Dawson et al., 1997). The absence of *Tsp-1* resulted in increased vascular and cellular proliferation within the islets, leading to significant glucose intolerance, reduced glucose-stimulated insulin secretion, and impaired (pro)insulin biosynthesis. These adverse effects in *Tsp-1*-deficient mice can be rescued by activating transforming growth factor β-1 (Tgfβ1) (Olerud et al., 2011; Drott et al., 2012; Crawford et al., 1998). Thus, Tsp-1 from ECs is crucial for β cell function through the activation of islet Tgfβ1. CD47, the receptor for TSP-1, is expressed in human islets (Erdem et al., 2023). Furthermore, Tsp-1 from ECs enhances β cell survival under lipotoxic (Cunha et al., 2016) and endoplasmic reticulum (ER) stress conditions (Cunha et al., 2017). In the embryo, Ctgf is highly expressed in the pancreatic ductal epithelium and ECs, where it is essential for β cell proliferation. In adulthood, Ctgf is primarily expressed in ECs of the pancreatic islet (Crawford et al., 2009). Treatment of islets with partial β cell damaged using Ctgf has been shown to enhance β cell proliferation and restore islet mass by 50% (Riley et al., 2015).

ECs can also produce basement proteins (extracellular matrix (ECM) components) (Nikolova et al., 2006; Kragl and Lammert, 2010), such as laminin and type IV collagen, which constitute the basement membrane of islets (Nikolova et al., 2006; Vartanian et al., 2009). In 2004, Kaido et al. demonstrated that islet ECs secrete type IV collagen, which interacts with integrin α1β1 expressed in both fetal and adult β cells, thereby enhancing insulin secretion (Kaido et al., 2004). Additionally, research by Nikolova and Sakhneny et al. revealed that laminins produced by ECs promoted β cell proliferation and increased expression of functional markers such as insulin 1 (Ins1), MAF bZIP transcription factor A (Mafa), and glucose transporter 2 (Glut2), as well as significantly improved glucose-stimulated insulin secretion (Nikolova et al., 2006; Sakhneny et al., 2021).

Berclaz et al. (2016) utilized extended-focus Fourier domain Optical Coherence Microscopy to create a label-free, three-dimensional imaging method for observing islets, which also enables a three-dimensional (3D) live-visualization of vascular network. With the availability of this technique, we anticipate better elucidating the collaboration between pancreatic islets and surrounding vasculature (Berclaz et al., 2016).

### 2.3 Role of islet ECs in pregnancy, aging, and diabetes

Adult islets in general have slow overall turnover rate. However, islet mass changes in response to altered metabolic demands. Increased islet mass is observed in pregnancy, obesity, or insulin resistance (Bonner-Weir, 2000; Sorenson and Brelje, 1997; Meier et al., 2006), conversely, aging is often associated with notable decline in β cell mass (Tschen et al., 2009; Rankin and Kushner, 2009; Kushner, 2013) (Figure 1C). In all these cases, ECs have been identified to play a part.

Maternal pancreatic islets enlarge during pregnancy (Sorenson and Brelje, 1997; Johansson et al., 2006b), in accordance with and likely result from elevated levels of placental prolactin, prolactin, and growth hormone. ECs not only mediate these drastic hormonal changes, but also support β cell proliferation, survival, and function through paracrine effects (Sorenson and Brelje, 1997; Brelje et al., 1993; Vasavada et al., 2000). It has been shown that EC proliferation occurs even before islet cell proliferation (Sorenson and Brelje, 1997; Johansson et al., 2006b). Johansson et al. found that proliferating ECs promote islet cell proliferation by secreting hepatocyte growth factor (Hgf), and reciprocally, islet cells produce vascular endothelial growth factor-A (Vegfa) and insulin to stimulate EC secretion (Johansson et al., 2006b).

Aging islets display reduced cell proliferation and regeneration, leading to decreased β cell mass (Tschen et al., 2009; Rankin and Kushner, 2009; Kushner, 2013; Chen et al., 2011). In 2021, Chen et al. showed a sparser vascular network within the aging mice islets, while the surrounding exocrine vessel density appeared unchanged. This study also identified a specific subset of ECs within the islets (CD31^+^ Emcn^hi^) that promote β cell proliferation. The CD31^+^ Emcn^hi^ ECs population declines with aging, which is attributed to increased expression of gap junction protein 1 (Gja1). Targeted ablation of *Gja1* restored EC proliferation and islet vessel density, consequently increasing β cell proliferation, β cell mass, and insulin production in aged islets (Chen et al., 2021). Taken together, it is plausible that decreased islet ECs disrupt islet homeostasis and lead to β cell loss with aging. In light of this study, targeted interventions aimed at restoring aging islet vasculature may prevent β cell loss, preserve islet function and potentially delay the onset of diabetes.

Diabetes can cause islet microvascular complications, including hyperplasia, basement membrane thickening, vessel dilation, rupture and impaired vasomotion (Mateus Gonçalves et al., 2023; Okajima et al., 2022). Under glycemic conditions, ECs within the pancreas express inflammatory and activation markers, which may lead to their dysfunction and consequently affect insulin secretion by β cells. The alterations observed in ECs in the context of diabetes have been comprehensively summarized in a previous review (Hogan and Hull, 2017), thus; they will not be reiterated in this discussion. The prevention of vascular damage could be beneficial for diabetes management. Studies have shown that metformin (Zou et al., 2022), rutin (David et al., 2023), and glycine supplementation (Wang B. et al., 2024) exhibit protective effects against vascular endothelium dysfunction in type 1 diabetes mellitus (T1DM). Further elucidation of the mechanisms by which pancreatic ECs support the survival and function of β cells may provide new therapeutic targets for diabetes treatment.

## 3 ECs for islet transplantation

**FIGURE 2:**
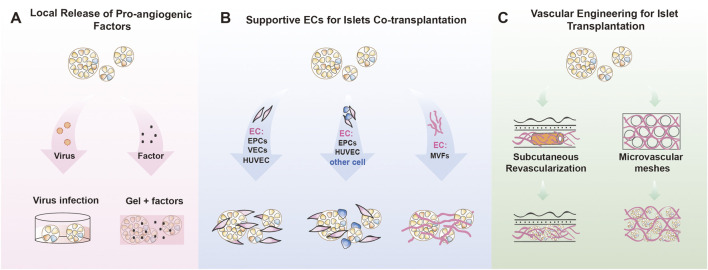
Endothelial cell for islet transplantation **(A)** Local factor release system: Vascularization of the graft is facilitated through the release of vascular growth factors. This can involve strategies such as viral-mediated overexpression of these factors in organoids or islets, or transplantation after coating the islets with a matrix gel or hydrogel infused with growth factors. **(B)** EC-islet co-TP system: ECs were co-transplanted with islets or organoids, including single ECs, multicellular types containing ECs, and functional microvascular co-transplantation systems. **(C)** Vascular Engineering for TP: Biomaterials were used to promote subcutaneous pre-vascularization in advance, or vascularization networks were constructed *in vitro* before islet or organoid transplantation. Endothelial cells (ECs); Transplantation (TP); Co-transplantation (co-TP); Endothelial progenitor cells (EPCs); Vascular endothelial cells (VECs); Human umbilical vein endothelial cells (HUVEC); Mesenchymal stem cells (MSCs); Human amniotic epithelial cells (hAECs); Human adipose derived stem cells (hADSCs); Microvascular fragments (MVFs).

Conventional treatment for diabetes relies primarily on oral hypoglycemic agents or daily insulin injections (Mathieu et al., 2021). However, these approaches can only ameliorate hyperglycemia temporarily but fail to restore autonomous glycemic control, until the first successful islet transplantation (Kobayashi, 2008; Bellin and Dunn, 2020; Powers, 2021; Shapiro et al., 2017; Rickels and Robertson, 2019; Paget et al., 2022; Wang Q. et al., 2024). Besides being a promising cure, islet transplantation has its own hitch, that is the unpredictable graft failure incidences. It is presumed that up to 60%–80% of islet cells may be lost within 48 h after transplantation (Davalli et al., 1995; Eriksson et al., 2009; Biarnes et al., 2002). To improve post-transplantation islet survival, many groups have tried accelerating revascularization of the transplanted islets (Figure 2).

### 3.1 Local release of pro-angiogenic factors

Employing local growth-promoting factors have been demonstrated to expedite the vascularization of transplanted grafts (Relevant studies are summarized in Table 1; Figure 2A). Vegf is a pivotal stimulator of angiogenesis and plays a crucial role in islet revascularization (Yancopoulos et al., 2000; Ferrara and Davis-Smyth, 1997). However, Vegf expression in islet cells significantly decline 2–3 days post-transplantation (Vasir et al., 2001). Studies aimed at overexpressing Vegf in transplanted islets have shown enhanced angiogenesis, increased vascular density, and improved graft blood flow (Lai et al., 2005; Zhang et al., 2004). Studies by Zhang et al. (2004) and Lai et al. (2005) demonstrated that Vegf overexpression not only promoted β cell proliferation and survival but also improved glycemic outcome in diabetic mice (Lai et al., 2005; Zhang et al., 2004). Furthermore, Golocheikine et al. (2010) reported that embedding mouse islets in a Vegf and Hgf enriched matrix gel has been reported to induce angiogenesis, reverse diabetes, and enhance the expression of adhesion molecules (Icam, Vcam) and signaling pathways (P-FAK, P-ERK1/2), supporting stable engraftment (Golocheikine et al., 2010). García A. J’s team developed an injectable VEGF-releasing polyethylene glycol hydrogel (Phelps et al., 2013; Phelps et al., 2015; Weaver et al., 2017; Weaver et al., 2018), which significantly enhanced islet vascularization and engraftment in T1DM mouse model. This VEGF-A protein hydrogel reduced the required number of islets by 40%, improved body weight and glucose responsiveness, and promoted intra-islet vascularization. In 2018, Gebe et al. introduced an islet implant scaffold, which can locally and controllably release Vegfa, and showed reduced early islet necrosis and improved graft performance (Gebe et al., 2018). Recent studies also highlight the role of endothelial cilia in regulating islet vascularization via the Vegfa/Vegfr2 pathway. Disruption of this pathway, as seen in *Bbs4* ^*−/−*^ islets, delays revascularization and impairs vascular permeability, ultimately hindering glucose delivery and islet function (Xiong et al., 2020).

**TABLE 1:** Summary of local release of pro-angiogenic factors.

References	The source and isolation of the EC	The source of the b-cell	Transplant model	EC function after TP (characterization/period observation)	Organoid/islet function after TP	Contributions
Zhang et al. (2004)	•Mouse •Primary •Autogenous EC	Mice fresh islets (Infected with Ad-VEGF)	•Virus infection •Kidney capsule •STZ-induced diabetic mice	•Vascular density ↑ •CD31^+^cell ↑ •EC marker: CD31 staining •16 days post-TP	•GTT↑ •GSIS (insulin secretion) ↑ •β Cell mass↑ •Glycemic control in diabetic mice (16 days) ↑	•Vegf production in islets stimulates graft angiogenesis and enhances islet revascularization •A novel strategy to accelerate islet revascularization and improve islet survival
Lai et al. (2005)	•Mouse •Primary •Autogenous EC	Mice fresh islets (RIP-VEGF-A mice)	•Virus infection •Diabetic mice •Hypoxic conditions	•EC sprout formation ↑ •Vascular density ↑ •Blood flow ↑ •EC marker: BS-1 staining	•Insulin secretion ↑ •β Cell mass (14 days) ↑ β Cell proliferation ↑ •Percentage of normoglycemia ↑	•A method for optimizing angiogenesis of islet transplants
Golocheikine et al. (2010)	•Mouse •Primary •Autogenous EC	Mice fresh islets	•A matrix gel containing VEGF and HGF •Subcutaneous transplantation •Diabetic mice	•Vascular density ↑ Stable blood vessel formation •EC marker: H&E staining 15 days post-TP	•Survival ↑ Suboptimal (250) islet equivalents could restore normoglycemia (30 days) •Islet apoptosis ↓ Insulin staining ↑	•Vegf and Hgf synergistically enhance angiogenesis after islet transplantation leading to stable engraftment
Phelps et al. (2013); Weaver et al. (2017); Weaver et al. (2018)	•Mouse •Primary •Autogenous EC	Mice fresh islets	•Engineered Vegf-releasing-PEG hydrogel •Hepatic portal vein/epididymal fat pad/S.c. site/small bowel •Diabetic mice	•Vascularization in extrahepatic transplant sites↑ •Intra-islet vascularization and engraftment↑ •EC marker: Lectin perfusion 2–4 weeks post-TP	•Insulin/C-peptide↑ •GTT ↑ •Superior glycemic function compared with hepatic portal vein transplantation •Reverse diabetes (<35 days) ↑	•Establishes a simple biomaterial strategy for islet transplantation to promote enhanced islet engraftment and function

**TABLE 1:** () Summary of local release of pro-angiogenic factors.

References	The source and isolation of the EC	The source of the b-cell	Transplant model	EC function after TP (characterization/ period observation)	Organoid/islet function after TP	Contributions
Gebe et al. (2018)	•Mouse •Primary Autogenous EC	Mice fresh islets	•A scaffolded islet implant-Vegf releasing •Subcutaneous transplantation •Diabetic mice	•Necrotic area ↓ •Vascularized area •Vascular density ↑ •EC marker: H&E staining •12 days post-TP	•Percentage of normoglycemia ↑ •Time to normoglycemia ↑ •GTT ↑ •Necrotic area ↓ <45 days	•The scaffolded islet implant can reverse diabetes in SC sites without prevascularization •Low-dose Vegf released from the implant helps reducing islet stress post-grafting
Su et al. (2007)	•Mouse •Primary •Autogenous EC	Mice fresh islets (transduction by Ad-Ang1)	•Kidney capsule •Diabetic mice	•Vascular density ↑ •Intra-islet vessels ↑ •EC marker: CD31 staining •30 days post-TP	•Islet survival ↑ •β Cell Mass ↑ Blood glucose ↓ •GSIS (insulin secretion) ↑ •GTT ↑ Reverse diabetes (<30 days)	•Ang-1 confers a cytoprotective effect on islets, enhancing islet engraftment and preserving functional islet mass in transplants
Karanth et al. (2021)	•Human •iPSCs-induced ECs	iPSCs induced islet organoid	•*In vitro*culture	•Generation of ECs and pericytes ↑ •EC marker: VE-cadherin/NG2 staining •22–29 days (*in vitro*)	•*In vitro* •Pancreatic progenitor markers ↑ •Islet hormone markers ↑ •Insulin release ↑ •GTT ↑	•First revealed that angiopoietins, including Ang-1 and Ang-2, enable iPSCs to generate islets with enhanced glucose responsiveness, a key feature of mature islets

Endothelial cells (ECs); Vascular endothelial growth factor (VEGF); Transplantation (TP); Streptozotocin (STZ); biotinylated Bandereira simplicifolia (BS-1); Glucose tolerance test (GTT); Glucose stimulated insulin release (GSIS); Poly ethylene glycol (PEG); Hepatocyte growth factor (Hgf); Hematoxylin and eosin (H&E); angiopoietin-1 (Ang-1); angiopoietin-2 (Ang-2); Induced pluripotent stem cells (iPSCs).

Besides Vegf/Vegfr2 signaling, other angiogenic pathways have also been explored. Su et al. (2007) investigated the role of angiopoietin-1 (Ang-1), a factor that promotes angiogenesis and inhibits apoptosis, in pancreatic islet transplantation. The study found that islet cells expressing Ang-1 exhibited significantly increased microvascular density, improved cell viability, and enhanced insulin secretion in response to glucose stimulation (Su et al., 2007). More recently, Karanth et al. (2021) revealed that angiopoietins, including Ang-1 and Ang-2, promote the generation of islets from induced pluripotent stem cells (iPSCs) with elevated glucose responsiveness and enhanced expression of all islet hormones. Islets stimulated by Ang-2 were capable of modulating insulin exocytosis in response to glucose stimulation through the dynamic process of actin filament polymerization and depolymerization. This regulation likely occurs via the CDC42-RAC1-gelsolin-mediated insulin secretion signaling pathway (Karanth et al., 2021). Overall, these findings indicate that pro-angiogenic growth factors can effectively enhance transplant vascularization, protect islet cells, and support the function of pancreatic islet transplants.

### 3.2 Supportive ECs for islets co-transplantation

Co-transplantation of islets with ECs has been explored in order to achieve rapid blood perfusion of transplanted islets with the host, thereby improving islet cell survival, function, and long-term graft viability (Relevant studies are summarized in Table 2) (Figure 2B).

**TABLE 2:** Summary of supportive ECs for islets co-transplantation.

References	The source and isolation of the EC	The source of the b-cell	Transplant model	EC function after TP (characterization/ period observation)	Organoid/islet function after TP	Contributions (Advantages-A, Limitations-L, Scalability-S)
2.2.1 I Co-transplantation of islets with ECs						
Kang et al. (2012)	•Human •EPCs •Primary (umbilical cord blood-derived (Hur et al., 2004))	Porcine islets	•Co-TP EPCs with Porcine islets •Kidney capsule •Diabetic mice (STZ)	•Rapid revascularization •Capillary/vessel density ↑ •Functional vessel ↑ •EC marker: VE-cadherin/Col IV/BS1-lectin staining •3/14/21/35 days after TP	•Better glycemic control •Reversal of diabetes (42 days) •β Cell proliferation ↑ Islet survival ↑	A: EPC Co-TP can enhance islet engraftment by rapid revascularization L: Only basic research S: Not strong
Quaranta et al. (2014)	•Rat •EPCs •Primary (BM-derived (Dobson et al., 1999))	Lewis rat islets	•Co-TP of EPCs with rat islet •Portal vein Diabetic rat (STZ)	•Endothelial thickness ↑ Vessel density ↑ •EC marker: PECAM-1 staining •30/180 days after TP	•Reversal of diabetes (180 days) •GTT ↑ Faster reversal of diabetes	A: Co-TP of EPCs and islets induced a stable rescue of glycemic control lasting for a significant fraction of the animal life span L: Only basic research S: Not strong
Oh et al. (2013); Penko et al. (2015)	•Mouse •EPCs •Primary (BM-derived (De Falco et al., 2004))	Fresh mice islets	•Co-TP BM-EPCs with islets •Kidney capsule Diabetic mice (STZ)	•Vessel number ↑ Graft revascularization ↑ •EC marker: CD31 staining •28 days after TP	•GTT ↑ Insulin creation ↑ •Diabetes reversal rate ↑ Faster reversal of diabetes •28 days observation	A: BM-EPCs Co-TP with islets can improve revascularization and organization of islet grafts L: Only basic research S: Not strong
Barba-Gutierrez et al. (2016)	•Mouse •VECs •Primary (Pancreas)	Mice islets	•Integration of islets coated with VECs •Kidney capsule Diabetic mice (STZ)	•CD31^+^cell ↑ •Functional integrated into isolated islets •EC marker: CD31 staining 3/7/10/30 days after TP	•A well-defined rounded shape •TUNEL-positive cells ↓ •Reversal of diabetes ↑ •30 days observation	A: VECs could be functionally integrated into isolated islets A: Islets that are coated with VECs increased their capacity to engraft L: Only basic research S: Not strong
Vlahos et al. (2017)	•Human •HUVEC •Primary (Lonza)	Fresh rat islets	•Islet embedded in •HUVEC coated modules •Diabetic mice (STZ)	•CD31^+^number ↑ •Intraislet vascularization ↑ •EC marker: CD31/α-SMA staining •7/14/21 days after TP	•Restoration of •Normoglycemia (21 days) •GTT ↑ •M2-like macrophages ↑	A: Sufficient to restore and maintain normoglycemia for 21 days A: Offer a suitable option for Subcutaneous islet transplantation L: Only basic research S: Not strong
2.2.2 Co-transplantation with other cell types						
Johansson et al. (2008)	•Human •MSCs; ECs •Primary (Dermis microvascular ECs)	Human islets	•Coating of islets with ECs and MSCs •*In vitro*culture	*In vitro* •EC coverage ↑ •ECs proliferation •Vascular sprouts ↑ •EC marker: CD31/UEA-1 staining •2–6 days (*in vitro*)	only *in vitro*culture •Insulin release ↑ •6 days observation	A: The composite EC-MSC islets as a means of improving islet engraftment L: Lack of long-term observation, only *in vitro*study S: Not strong
Takebe et al. (2015)	•Human •MSCs; HUVEC •Primary (Lonza)	MIN6	•Generation of 3D vascularized organ buds *in vitro* •Kidney capsule Diabetic mice	•Rapid reperfusion ↑ •Vascularity ↑ •Functional microvascular networks ↑ •EC marker: CD31/Dextran staining •10 days after TP	•Restoration of •Normoglycemia (10 days) •Rapid normalization of body weight and blood glucose levels	A: A method to make organoids by mixing epithelial, mesenchymal and vascular cells L: Lack of long-term observation S: Not strong
Takahashi et al. (2018a)	•Human HUVEC; MSCs •Primary (Lonza)	Human and mice islets	•Self-Condensation Culture: HUVEC, MSCs and islet (3 days) •Kidney capsule Diabetic mice	•Rapidly re-perfused *in vivo* •Enhances engraftment •EC marker: CD31/Dextran staining •7/14/35 days after TP	•Restoration of Normoglycemia (35 days) •Therapeutic Capability ↑ •Insulin secret ↑ •GTT ↑	A: Self-condensation culture enables endothelialization of diverse tissue fragments A: Vascularized islet transplant enhances therapeutic potential against diabetes L: Lack of long-term observation L: The use of two distinct stromal progenitors especially about HUVEC S: Not strong
Soltanian et al. (2019)	•Human •ES-EC; ES-MSC •Cell line	ES-PP	•3D‐printed tissue trapper device •Intraperitoneal •transplantation Nude mice	•Vessel area ↑ •Number of vessels ↑ •EC marker: •CD31/CD144/vWF staining (*in vitro*)	•Insulin^+^cell ↑ •β cell maturation ↑ •C-peptide release ↑ •Transplantation for 90 days	A: The first report showing considerable capacity of hPSC derivatives (ES‐PPs, ES‐ECs, and ES‐MSCs) for formation of functional PO. L: Only basic research S: Not strong
Yoshihara et al. (2020)	•Human •hADSCs; HUVECs •Primary	iPSCs	•Form organ-like and vascular •structures in 3D •Kidney capsules •Diabetic mice (STZ)	N	•Restoration of Normoglycemia (40 days) •Rapidly reestablish glucose homeostasis •Insulin secretion *in vitro*↑ •GTT	A: HILOs contain endocrine-like cell types that, upon transplantation, rapidly reestablish glucose homeostasis in diabetic mice L: Lack of long-term observation S: Not strong
Wassmer et al. (2021)	•Human •hAECs; HUVECs Baudin et al., 2007	Rat islet cells	•Aggregation of hAECs and HUVECs with islets *in vitro*(5 days) •Epididymal fat pad Diabetic mice (STZ)	•Better engraftment and improved vascularization *in vivo* •Angiogenesis factor (Vegf-a) •EC marker: CD34/Lectin staining •30 days after TP	•Islet function *in vitro*↑ •β cell function (Glp-1r, Pdx1) ↑ •Glycemic Control •30 days observation	A: Better engraftment and improved vascularization *in vivo*in a murine model L: Lack of long-term observation L: Difficult to generate large numbers of uniform and functional prevascularized islet organoids S: Not strong
Naqvi and Naqvi (2023)	•Mouse •MSCs; EPCs •Primary (BM-derived)	Fresh mice islets	•Self-condensation of MSCs and EPCs with mice islets •Omental pouch •Diabetic mice (STZ)	•CD31^+^cell ↑ •Mice endothelial markers expressed •Induction of angiogenic factors •EC marker: CD144/CD31/CD106 staining •7days (*in vitro*)	•Restoration of normoglycemia •C-peptide ↑ •GTT ↑ •100 days observation	A: Co-TP of MSCs and EPCs along with islet to improvise the maximum survival time of mice islets to restore normoglycemia L: Only basic research S: Not strong
2.2.3 Co-transplantation with MVFs						
Salamone et al. (2021)	•Rat •MVFs •Primary (Adipose tissue)	Rat islets	•Coculture into Type-I collagen hydrogel •*In vitro*culture (28 days)	•Angiogenesis factors ↑ •Forming tubes, branches, and entire capillary networks •EC marker: CD31/CD90 staining •8 days (*in vitro*)	only *in vitro*culture •A vascularized islet •Basal lamina surrounded islet •GSIS – •Cultured for 8 days *in vitro*	A: Ad-MVF–islet of Langerhans coculture to improve the islets vascularization L: Only *in vitro*study S: Not strong
Nalbach et al. (2021a)	•Mice •MVFs Primary (Epididymal fat pads Frueh et al., 2017b)	Mice islets/Human islets	•Liquid overlay coculture (Prevascularized islet organoids) •Kidney capsule Diabetic mice (STZ)	•Microvasculature •angiogenic activity •functional capillary density ↑ •EC marker: CD31/Dextran staining •14/28 days	•Organoid viability ↑ •Insulin secretion ↑ •GTT ↑ •Reversal of diabetes (28 days)	A: The prevascularized islet organoids are rapidly blood-perfused and interconnected host vessels A: A lower number of islet grafts are required to restore normoglycemia in diabetic mice L: This technique is time-consuming S: Difficult to implement into clinical practice
Aghazadeh et al. (2021)	•Rat •MVFs •Primary (Epididymal adipose tissue)	HESC-derived pancreatic cells/Fresh human islets	•Co-transplantation of islets and MVs in collagens I •Subcutaneous transplantation •Diabetic mice (STZ)	•Vessel perfusion ↑ •EC number ↑ •EC marker: CD31/Lectin/αSMA staining •1/7/14 weeks after TP	•Blood glucose ↓ •GTT ↑ •Islet survival ↑ β cell maturation ↑ •Long-term glycemic control Reversal of diabetes (15 weeks)	A: Ready-made MVs are superior to single endothelial cells in TP A: Ready-made MVs improve hESC-derived pancreatic cells and human islet engraftment L: The maturation of β cells *in vivo* S: Has certain clinical translational potential
Wrublewsky et al. (2022)	•Mouse •MVFs •Primary (Epididymal adipose tissue)	Fresh mice islets	•Co-transplantation of islets and MVs •Kidney capsule/subcutaneous transplantation •Diabetic mice (STZ)	•CD31^+^cell ↑ •EC marker: CD31 staining 28/105 days after TP	•Islet survival ↑ •β Cell Mass ↑ •Blood glucose ↓ •GSIS ↑ Reversal of diabetes (105 days)	A: A low number of subcutaneously transplanted islets can reverse diabetic mice L: only basic research S: Not strong

Endothelial cells (ECs); Transplantation (TP); Streptozotocin (STZ); biotinylated Bandereira simplicifolia (BS-1); Bone marrow (BM); Transplantation (TP); Bone marrow-derived early endothelial progenitor cells (BM-EPCs); Endothelial progenitor cells (EPCs); Glucose tolerance test (GTT); Vascular endothelial cells (VECs); human umbilical vein Endothelial cells (HUVECs); Ulex europeaus agglutinin-1 (UEA-1); Human amniotic epithelial cells (hAECs); Mesenchymal stem cells (MSCs); von Willebrand factor (vWF); Induced pluripotent stem cells (iPSCs); Microvascular fragments (MVFs); Embryonic stem (ES) cell-derived pancreatic progenitors (ES-PP); Glucose stimulated insulin release (GSIS).

#### 3.2.1 Co-transplantation of islets with ECs

Bone marrow-derived endothelial progenitor cells (EPCs) have been used in multiple studies to facilitate the reconstruction of the graft’s vascular network (Krenning et al., 2009; Garikipati and Kishore, 2017; Critser and Yoder, 2010) (Relevant studies are summarized in Table 2). Kang et al. (2012) co-transplanted porcine islets with human umbilical cord blood-derived EPCs into diabetic nude mice (Kang et al., 2012), resulting in the accelerated normalization of blood glucose levels within approximately 11 days (islet-only group failed to achieve normoglycemia over 5 weeks) and higher serum porcine insulin levels. The authors attributed this significant improvement to increased Vegf/Hgf secretion and basement membrane production from the co-transplanted umbilical cord blood-derived EPCs (Kang et al., 2012).

Similarly, Quaranta et al. (2014) co-transplanted islets with EPCs into the portal vein of diabetic rats, achieving sustained normal blood glucose levels for up to 180 days, while the control group remained below the diabetic threshold throughout the observation period (Quaranta et al., 2014). Further studies by Oh et al. (2013) and Penko et al. (2015) demonstrated that co-transplanting mouse islets with bone marrow-derived EPCs into the renal capsule of diabetic mice improved glucose tolerance, serum insulin levels, and diabetes reversal rates compared to islet transplantation alone (Oh et al., 2013; Penko et al., 2015). This approach also increased vascular density from both donor and recipient sources (Oh et al., 2013). Overall, co-transplantation with EPCs showed improved islet function. *In vitro* studies have shown that EPCs reduced the expression of connexin 36 on β cell and enhanced glucose-stimulated insulin release (Penko et al., 2015).

Alternative EC sources for co-transplantation with islets have also been explored. In 2016, Barba-Gutierrez et al. encapsulated mouse islets with pancreatic-derived vascular ECs and transplanted them into streptozotocin-induced diabetic mice. This approach improved islet structures maintenance, enhanced vessel integration, reduced TUNEL^+^ apoptotic cells, and accelerated normoglycemia after transplantation compared to islets alone (Barba-Gutierrez et al., 2016). Similarly, Vlahos et al. (2017) embedded rat islets within collagen modules containing human umbilical vein ECs (HUVECs) and transplanted them subcutaneously into diabetic mice. This approach resulted in sustained glucose normalization for up to 21 days, a result not achieved with an equivalent number of free islets, and improved islet revascularization and host integration. Mechanistically, collagen-embedded islets promoted a shift toward an M2-like macrophage response, which is associated with vascularization and tissue remodeling (Vlahos et al., 2017).

#### 3.2.2 Co-transplantation with other cell types

In addition to ECs, the co-transplantation of islets with other supportive cells has also been explored (Relevant studies are summarized in Table 2). In 2008, Johansson et al. co-cultured human mesenchymal stem cells (MSCs) and dermal microvascular ECs with human islets *in vitro*, promoting the formation of vessel-like structures before transplantation, which potentially enhanced islet engraftment success (Johansson et al., 2008). In 2014, Taniguchi’s team developed a dynamic self-condensation technique to create tissue organoids from dissociated organ progenitor cells in the presence of ECs and MSCs (Takebe et al., 2014). Subsequently, Takahashi et al. (2018) cocultured dissociated murine β cell lines (MIN6), human and murine islets with HUVECs and human MSCs to form self-organizing vascularized islets (Takahashi et al., 2018a; Takebe et al., 2015; Takahashi et al., 2018b). This method not only improved cell survival and functionality *in vitro* but also significantly enhanced therapeutic outcomes post-transplantation (Takahashi et al., 2018b; Homma et al., 2023).

In 2019, Soltanian reported the generation of a type of pancreatic organoids by assembling various cell types derived from embryonic stem (ES) cells, including human ES-derived PDX1^+^ cells, MSCs, and ECs. The human pancreatic organoids exhibited enhanced vascularization, characterized by greater vessel density and quantity, a higher count of Insulin^+^ cells, and improved secretion of human C-peptide, with glycemic effects persisting for 90 days post-transplantation (Soltanian et al., 2019). In 2020, Yoshihara et al. used human iPSCs to generate human islet-like organoids by incorporating human adipose-derived stem cells (hADSCs) and HUVECs into multicellular spheroids. These organoids, containing insulin-producing cells, were transplanted into diabetic mice and maintained glucose homeostasis for approximately 40 days, with similar effects to human islets (Yoshihara et al., 2020). In 2022, Wassmer et al. developed and transplanted prevascularized islet-like clusters made from rat pancreatic islets, human amniotic epithelial cells (hAECs), and HUVECs. Compared to fresh islets, these pre-vascularized islet-like clusters improved engraftment and vascularization in mouse models. This improvement is likely attributed to the interaction between hAECs, HUVECs, and islet cells, through the upregulation of angiogenesis-promoting genes (Vegfa) and β cell function-related genes (Glp1r, Pdx1) (Wassmer et al., 2021). More recently, (Naqvi and Naqvi, 2023) demonstrated improved glucose clearance by transplanting a mixture of mouse MSCs, EPCs, and islets (Naqvi and Naqvi, 2023).

Collectively, these studies suggest that creating a pre-vascularized niche with ECs and other supportive cells can significantly improve glycemic function of transplanted islets or islet-like organoids (Takahashi et al., 2018b).

#### 3.2.3 Co-transplantation with microvascular fragments (MVFs)

The above-mentioned studies typically use only ECs for co-transplantation with islets; still, on average it takes over a week to achieve integration with the host (Laschke and Menger, 2016). In contrast, MVFs isolated from adipose tissue retain intact endothelialized lumens and are covered with vessel mural cells, providing a rich source of angiogenic factors. It has been shown that MVFs can rapidly reassemble into functional microvascular networks and integrate with host blood vessels, ensuring rapid and sustained blood perfusion of the graft (Shepherd et al., 2004; Sun et al., 2020; Laschke and Menger, 2015; Nalbach et al., 2021a). Hence, MVFs appear to be potent vascularization units for tissue engineering and regenerative medicine (Laschke and Menger, 2015; Nalbach et al., 2021a; Laschke and Menger, 2022; Sun et al., 2022; Frueh et al., 2017a; Frueh et al., 2017b) (Relevant studies are summarized in Table 2).

In 2021, Nalbach et al. and Salamone et al. successfully generated vascularized pancreatic organoids by co-culturing islet cells with functional MVFs *in vitro* (Nalbach et al., 2021b; Salamone et al., 2021). These vascularized pancreatic organoids exhibited significantly enhanced angiogenic capacity and quickly integrated with host blood vessels as early as day 3, mediated by paracrine signaling between β cells and ECs through Hgf/c-Met/Ho-1 pathway (Nalbach et al., 2021b). In the same year, Aghazadeh et al. found that co-transplantation of rat MVFs markedly improved cell survival and glucose responsiveness of human islets and human embryonic stem cell (hESC)-derived pancreatic organoids in three different T1DM mouse models, leading to a rapid reversal of diabetes (Sun et al., 2022; Aghazadeh et al., 2021; Powers and Brissova, 2021; Hoesli and Kieffer, 2021). Similarly, Wrublewsky (2022) and Nalbach et al. (2021) co-transplanted mouse islets and adipose tissue-derived MVFs into recipient mice. This combined approach accelerated blood glucose recovery in diabetic mice compared to islet transplantation alone, allowing effective glucose reduction with fewer islets (Nalbach et al., 2021b; Wrublewsky et al., 2022).

Studies have shown that co-transplanting islets with MVFs accelerates vascular integration with the host, reducing graft ischemic time and enhancing transplantation success. This approach holds promise for using recipient-derived adipose tissue microvasculature to potentially decrease the required islet mass, improve graft survival, and expedite the achievement of normoglycemia. However, a key challenge remains obtaining sufficient quantities of host-derived microvasculature for clinical application.

### 3.3 Vascular engineering for islet transplantation

The standard method for islet transplantation involves infusing islets into the hepatic portal vein. However, many donor islets are lost due to immediate blood-mediated inflammatory response (IBMIR) and ischemia (Pepper et al., 2018; Shapiro et al., 2017; Naziruddin et al., 2014). Patients often require multiple islet infusions to achieve insulin independence. Finding a minimally invasive site that can support the survival of transplanted islets is necessary. Subcutaneous pancreatic islet transplantation seems like a promising option but is limited by poor vascularization, which fails to provide adequate oxygen and nutrients for islet survival and function (Vlahos et al., 2017; Lacy et al., 1991), thereby limiting its application. With rapid advancements in biomaterials and bioengineering strategies, researchers are exploring the possibility of using these to address vascular deficiencies in subcutaneous islet implantation (Relevant studies are summarized in Supplementary Table 1) (Figure 2C).

#### 3.3.1 Utilizing biomaterials to promote subcutaneous neovascularization

Promoting subcutaneous vascularization prior to islet transplantation is expected to reduce the time required for circulation connection. Initially, research has focused on using biomaterials to enhance neovascularization at the subcutaneous site. In 2017, Mahou et al. developed a semi-interpenetrating polymer network (SIPN) that facilitated blood vessel growth at the subcutaneous implantation site. Islets embedded in SIPN remained viable and responsive to glucose stimulation *in vitro*, and transplanted diabetic mouse models showed a progressive return to normoglycemia (Mahou et al., 2017). Two other studies aimed to ensure islet transplantation success by employing basic fibroblast growth factor (bFGF) to induce neovascularization at subcutaneous sites. In 2000 and 2001, Kawakami et al. implanted a bFGF-releasing device 1 week before transplantation, resulting in a well-vascularized capsule and maintenance of normoglycemia for over a month post-transplantation (Kawakami et al., 2000; Kawakami et al., 2001). Similarly, in 2014, agarose rods containing bFGF and heparin were implanted 1 week before surgery in dorsal subcutaneous sites. After removing the rods, islets were transplanted into the pre-vascularized sites, quickly reversing hyperglycemia in diabetic rats within 1–3 days. Notably, allogeneic islets transplanted to these pre-vascularized sites demonstrated long-term graft survival and function compared to those transplanted into the hepatic portal vein (Luan and Iwata, 2014). These findings suggest the pre-vascularizing subcutaneous sites prior to islet transplantation can improve graft function by ensuring a sufficient blood supply, thereby reducing the time required for islet engraftment and even reducing immune rejection.

#### 3.3.2 Utilizing bio-devices to promote subcutaneous neovascularization

The use of bio-devices to promote subcutaneous neovascularization has also been explored. For instance, Craig et al. (2005) and Pileggi et al. (2006) pre-implanted a cylindrical stainless-steel mesh to facilitate the formation of blood vessels and connective tissue around the device (Craig et al., 2005; Pileggi et al., 2006). When syngeneic islets were transplanted into these vascularized devices, they restored normal blood glucose levels and maintained long-term functionality, comparable to portal vein transplantation (Craig et al., 2005; Pileggi et al., 2006). Upon removal of the device, hyperglycemia was observed, while the preserved islets and intense vascular network were evident in the retrieved grafts (Pileggi et al., 2006). In 2005, Andrew et al. used a subcutaneous cell pouch device implanted 3–4 weeks prior to induce neovascularized tissue chambers. Islets transplanted into these chambers in mice restored blood glucose control rapidly and responded well to glucose challenges, similar to intrarenal subcapsular islet transplantation (Andrew R Pepper et al., 2015).

Recent studies suggest that the use of biological devices can prevent immune rejection in subcutaneous transplantation. Sorenby et al. (2008) utilized TheraCyte immunoprotective devices implanted subcutaneously to induce vascularization. This approach reduced the required dose of encapsulated islets by approximately 10-fold, while also achieving normoglycemia comparable to sole islets kidney encapsulation (Sorenby et al., 2008). Similarly, Smink et al. (2017) described a novel pre-vascularized, subcutaneously implanted, retrievable poly (D, L-lactide-co-ε-caprolactone) scaffold, which protected the viability and function of islets, enhancing their engraftment in subcutaneous sites (Smink et al., 2017). In 2020, Liu et al. modified a pre-vascularized tissue-engineered chamber (TEC) to improve the viability and function of the seeded islets *in vivo* by providing a microvascular network before transplantation (Liu et al., 2020). Notably, xenogeneic islets within TECs maintained long-term recipient-specific immune tolerance (Liu et al., 2020). Recently, Shapiro’s team (2023) implanted a hollow nylon catheter into the subcutaneous space, inducing a controlled inflammatory response in the host that created a vascularized pocket several weeks later (Pepper et al., 2015). After catheter withdrawal, a thread-like alginate-based islet-encapsulation device was transplanted into this modified pocket (An et al., 2018). This SHEATH system enabled sustained diabetes reversal, significantly increasing oxygenation, physiological glucose responsiveness, and islet survival rates. Moreover, it allowed for *in situ* replacement of damaged devices and rapid restoration of normal blood glucose levels. This device system holds promise for facilitating the clinical translation of immunosuppression-free subcutaneous islet transplantation (Wang et al., 2023). In 2019, Song et al. developed a functional microvascular mesh using an anchored self-assembly (ASA) strategy. These microvascular meshes significantly enhanced the vascularization of rat islets transplanted under the skin and achieved a 3-month correction of diabetic mice (Song et al., 2019).

These results suggest that subcutaneous vascularization devices, in combination with various biomaterials, serve as promising therapeutic assistance to evade immune attack. However, these studies rely on mouse models only, and lack long-term observation. Supportive data for preparing clinical translation such as extent of vascularization, immune infiltration, as well as long-term cell survival and function stability. Most importantly, the scalability of the current technique remains unknown. So far, only Shapiro’s team assessed the scalability of their SHEATH system using a minipig model, demonstrating that the implantation, removal, and replacement are minimally invasive, technically feasible, and scalable, for clinical application (Wang et al., 2023).

## 4 ECs in stem cell-derived β cells and islet organoids co-culture

The general implementation of islet transplantation is hindered by the scarcity of organ donors as well as laborious isolation procedures. One approach to solve the islet supply shortage is to obtain an adequate quantity of functional insulin-secreting cells *ex vivo*.

The ongoing advancements in differentiating functional β cells from human pluripotent stem cells (hPSCs) and islet organoid technologies hold immense potential to revolutionize traditional diabetes treatments. Over the past decade, researchers have investigated and optimized various protocols for expanding and inducing pancreatic β cells *ex vivo*. These methodologies encompass the utilization of ESCs, iPSCs, pancreatic islet progenitor cells derived from embryonic or adult tissues, and re-differentiation or trans-differentiation from exocrine cells (Hogrebe et al., 2023; de Klerk and Hebrok, 2021; Yu and Xu, 2020; Casamitjana et al., 2022; Jiang et al., 2022; Zhou et al., 2008; Al-Hasani et al., 2024; Li et al., 2014; Pagliuca et al., 2014; Rezania et al., 2014; Wang et al., 2020). At the same time, incorporation of ECs in coculture systems has been shown to significantly improve cell yields and organoid functions (Relevant studies are summarized in Table 3).

**TABLE 3:** Summary of Endothelial Cell Coculture Used in the Generation of β-like Cells and Islet Organoids.

References	The source and isolation of the EC	The source of the β cell	Cultivation method	Organoids’ function	EC function (characterization/period observation)	Contributions
3.1 Formation of Pseudoislets from β-Cell Lines and ECs						
Penko et al. (2011)	•Rat •EPCs Primary (Bonder et al., 2009)	Rat islet cells	•Re-aggregation technique •An embryoid body forming medium (BPEL) and a spin protocol <7 days	•Viability of cell ↑ •Islet function ↑ •GSIS (insulin secretion) ↑	•Tube formation *in vitro* •Uptake of acetylated low density lipoprotein •EC marker: Vegfr2, CD31 and Vcam1 were expressed •14 days (*in vitro*)	•Mosaic pseudoislets were formed by pancreatic β cells and EPC *in vitro*for the first time •Mosaic pseudoislets maintained function *in vitro*
Urbanczyk et al. (2020)	•Human •HUVEC •Primary (PromoCell)	EndoC-βH3	•Magnetic levitation to create two-layered heterotypic spheroids •“1:1”; “ECs inside”; “β -cells inside” •25 days	•CD31^+^ cell ↑ •Cell viability ↑ •GSIS ↑ •β cells surrounded by HUVECs express significantly more E-cadherin	•EC marker: CD31 staining •2/5 days (*in vitro*)	•The first study that introduce *in vitro* testing systems based on human cell lines and ECs •Investigate the impact of the spatial distribution on cocultures of human-cells and ECs
Porter et al. (2023)	•Human •HUVEC •Primary (Lonza)	EndoC-βH5	•100% β cells, 1: 1 β cell/endothelial, and 1: 3 β cell/endothelial •7 days	•GSIS ↑ Metabolic activity ↑	•EC marker: CD31 staining •2 days (*in vitro*)	•Ratio of β cell/EC affects insulin release
Wang et al. (2024a)	•Human •HUVEC Primary (Lonza)	MIN6	•Transwell •Min6 cells to HUVEC cells was 1:2 •3 days	•GSIS ↑ •β cell markers ↑ •After TP •GTT ↑	•EC marker: CD31 staining	•ECs promote islet cell function through the BTC-EGFR-JAK/STAT signaling pathway
Wieland et al. (2021)	•Human •HUVEC •Primary (Lonza)	INS1E	•α:β (1:14), α:β:EC (1:9:5), 1× α:β:EC (1:9:2), 2× α:β:EC (1:9:5) and 4× α:β:EC (1:9:20) •5 days	•Oxidative Stress ↓	N	•Provide important insights into the roles of α and ECs in protecting against oxidative stress
Amin et al. (2021)	•Human •Immortalized HUVECs (EA.hy926) •Cell line	MIN6	•ECs pre-treated with different concentrations RA •thapsigargin-induced ER stressed pancreatic β-cells	•Nrf2 ↑ •Heme oxygenase-1 (HO-1) and NADPH-quinone oxidoreductase-1 (NQO-1) ↑ •ER stress ↓ •Glucose-regulated protein (GRP) 78 and C/ERB homologous protein (CHOP) ↓	•Levels of growth factors like VEGF, EGF and FGF reduced in ER stress-induced ECs; treatment with RA restored their expression	•RA sensitized ECs confer protection to β cells
Spelios, (2018)	•Mouse •MS1 Cell line	Human EndoC- H1-cells	•Uncoated 6-well tissue culture plates •Self-Condensation 5–14 days	•Cell viability ↑ •Insulin expression ↑ •Insulin secretion ↑ •β cell associated Gene ↑ •GLP1/phosphokinase A expression ↑	•EC marker: BS1 staining	•The organoids increase endocrine marker expression and insulin secretion •The organoids increase GLP-1 receptor and phosphokinase A expression
Daniel et al. (2021)	•Rat •IMEC •Primary (Islets microcapillary)	INS1E	•Condition medium from IMEC •1 day	•GSIS ↑	•Glycolytic enzyme triosephosphate isomerase was secreted from ECs	•An effective paracrine interaction exists between IMEC and β cells and modulates glucose-induced insulin secretion via TPI-sulfonylurea receptor-KATP channel (SUR1-Kir6.2) complex attenuating interactions
Jung et al. (2022)	•Mouse •MS1 Cell line	βTC6	•1 day	•HuD downregulation in βTC6 cells inhibited the growth and migration of MS1 cells	•EC marker: Pecam1 staining	•HuD has the potential to regulate the crosstalk between β cells and islet endothelial cells by regulating Endostatin and Serpin E1 expression, thereby contributing to the maintenance of homeostasis in the islet microenvironment
3.2 Induction of Pancreatic β-cells from hPSCs in the Presence of ECs						
Talavera-Adame et al. (2011); Talavera-Adame et al. (2016)	•Human •HMEC •Cell line	EBs derived from hPSC	•Self-condensation culture system •Collagen–laminin gels •About 7 passages	•β cell marker ↑ •Expansion *in vitro* ↑ •GSIS ↑(C-peptide and insulin secretion) •After TP •C-peptide ↑ •β cells survival↑ •Blood glucose level ↓ •Long-term function and existence (>120 days)	•EC marker: CD31 staining	•Cocultured EBs had a higher expression of mature cells markers and enhanced insulin secretion *in vitro* •Cocultured EBs remain long-term existence
Augsornworawat et al. (2019)	•Human •HUVEC Cell line	hESC	•Hydrogel platform (aggregates) •Three-dimensional assemblies 1–20 days	•Viability of β cell ↑ •GSIS ↑ •(insulin secretion) •β cell marker ↑	•EC maker: CD31 expression •The assembled tubule network was formed	•The organoids express β cell and other endocrine markers and are functional
Candiello et al. (2018)	•Human •HUVEC Cell line	hESC	•A hydrogel-based platform •Spontaneous aggregation About 7 days	•β cell marker (PDX1/NKX6.1)↑ •Insulin Release ↑ Islet maturation ↑	N	•Organoids express β cell markers •Organoids secrete functional insulin production in response to glucose challenge
Chmielowiec et al. (2022)	•Human •MSC and EC (fetal pancreas, duodenum, and spleen), HUVEC and MS1 •Primary cell and cell line	hESC	•A hydrogel-based platform •A stepwise manner About 14 days	•C-peptide + cell ↑ •Ins + cell ↑ •C-peptide secretion ↑ GSIS ↑	•EC markers: PECAM1, CFIII, FLK1, CDH5, ICAM, VWF were expressed •The endothelial gene expression decreased after 8 passages	•This study shows that pancreatic microenvironment-derived factors can mimic *in vivo*conditions *in vitro*to generate authentic β cells for translational applications WNT5A activates the non-canonical (JNK/c-JUN) WNT signaling and works with Gremlin1 to inhibit the BMP pathway during β cell maturation
Yang et al. (2024)	•Human •hESC-derived Cell line	Differentiated MEL-1I^*NS/GFP*^hESCs into pancreatic endocrine cells	•VMI organoids, +differentiated H9 hESCs toward macrophages •14 days	•GSIS ↑ •β cell markers ↑ •β cell proliferation, survival, and maturation ↑	•EC marker: CD31 staining (*in vitro*: 7/14 days) •Uptake of acetylated low-density lipoprotein	•Proinflammatory macrophages induce β cell pyroptosis through the TNFSF12-TNFRSF12A pathway
3.3 ECs Assist in Establishing Pancreatic Organoids from Adult Stem Cells						
Song et al. (2009)	•Rat •Thoracic aorta EC •Primary	Rat islets	•Cocultures with thoracic aorta •7 days	•Islet viability >90% Insulin release ↑	•The morphology showed a paved monolayer with a periplast showing a characteristic buffy positive reaction	•Coculture of freshly isolated rat islets with ECs enhances their survival and function in vitro
Lehmann et al. (2019)	•Mouse •pancreatic blood vessels •Primary	mice islets	•3D culture system •Embedd in Rat tail type 1 collagen •8 days	•Islets remained viable •Insulin expression↑ •Slight growth of ß-cells •Microvessels were encapsulate islets	•EC marker: CD31expression (*in vitro*, 8 days)	•First isolation and culture of pancreatic blood vessels •A novel explant culture model for angiogenesis and tissue engineering research
Wang et al. (2020); Wang et al. (2022)	•Mouse •ECs •Primary (mammary gland/skin)	Procr + endocrine progenitor cells	•Re-aggregation •2.5D Matrigel culture system •Long-term expansion (>100 days)	•Long-term expansion (>100 days) •Maturation *in vitro* •C-peptide and insulin secretion ↑ •Glucose responsive ↑ •After TP Maturation •GTT ↑ Insulin secretion ↑ Reverse diabetes (<125 days)	•EC marker:CD31 staining (*in vitro*, 28 days) •Endothelial networks were formed •ECs require fresh isolation after each passage	•First identification of pancreatic islet progenitors •Long-term expansion and maturation in vitro •The organoids can reverse diabetes in vivo
3.4 Development of Engineered Models for Coculture of EC and Islet Cells						
Everwien et al. (2020)	•Rat •RAEC •Cell line	Primary rat islets	•Decellularization of rat liver and re-endothelialization •3 days	•GSIS ↑	N	•Develop a bioengineered platform to generate implantable and functional endocrine Neo-Pancreases
Rambol et al. (2020)	•Human •HUVEC •Cell line	Rat islets	•A commercially available microfluidic platform to generate perfusable microvascular networks •6 days	•Microvascular networks form perfusable lumens Microvascular network recruitment to islets	•Interconnected networks were formed Microvascular networks formed perfusable lumens	•A commercially available mi- crofluidic platform to generate perfusable microvascular networks, and by incorporating pancreatic islet.
Seo et al. (2020)	•Mouse •MS1 Cell line	Min6	•Cell-laden collagen sheet 15 days	•Blood vessel-like structure formation Cell viability of over 85% for about 2 weeks Insulin secretion ↑	•A monolayer of a vessel-like structure was formed	•A geometrically controlled pancreatic pseudo-tissue model •The MIN6 cells formed islet-like clusters surrounded by an endothelial MS1 cell monolayer, and show higher insulin secretion
Wassmer et al. (2021)	•Human •HUVEC •Primary Baudin et al., (2007)	Primary rat islets	•+Human amniotic epithelial cells •ICs, HUVECs and hAECs at a ratio of 5:4:1 4 days	•GSIS ↑ •After TP •Glycemic control ↑ Graft revascularization ↑	•Morphologically, cells displayed typical elliptic shape •EC markers vWF/CD31/CD144 staining •Over a period of 6 h	•The possibility of adding a selected source of endothelial cells for the neo-vascularization of insulin-scereting grafts may also allow implementation of β cell replacement therapies in more favourable transplantation sites than the liver
Zbinden et al. (2021)	•Human •HUVEC Primary (PromoCell)	INS1E	8 days •Encapsulated in COL1	•GSIS ↑ •Basement membrane proteins ↑	•EC marker: CD31 staining	•Providing ECs into the COL1 hydrogel improves β cell response as well as the expression of relevant BM proteins
Hospodiuk-Karwowski et al. (2022)	•Rat •RHMVEC Cell line	β-TC3	•A hydrogel-based perfusable	•Cell viability ↑ •Sprouting number and length ↑ •Insulin secretion ↑ •VEGF-A, Endothelin-1, and NOS3 expression ↑	•EC marker: CD31 staining •The endothelial cell-related genes VEGF-A, Endothelin-1, and NOS3 were expressed	•The device with the pancreatic-like spheroids was 3D bioprintable and perfusable
Quintard et al. (2024)	•Human •HUVEC Cell line	EndoC-βH5	•+Fibroblasts •A ratio of 6:1:1 (EndoC-βH5:FMA73:HUVEC-RFP, 10,000 cells per well) 30 days	•GSIS ↑	•Vessel-like structures were formed, covered by pericytes and smooth muscle cells, and exhibited the expression of tight junction protein ZO-1 and adherens junction protein VE-cadherin •EC marker: CD31 staining (*in vitro*,1–30 days)	•This microphysiological system represents a viable organ-on-chip model to vascularize diverse biological 3D tissues and sets the stage to establish organoid perfusions using advanced microfluidics

Bone marrow (BM); Transplantation (TP); Endothelial progenitor cells (EPCs); Embryoid bodies (EBs); Human microvascular endothelial cells (HMECs); Human pluripotent stem cells (hPSCs); human embryonic stem cells (hESCs); Mouse pancreatic islet endothelial cell line (MS1); Endothelial cells (ECs); Dissociated murine β cells (MIN6); Glucose stimulated insulin release (GSIS); Human umbilical vein endothelial cells (HUVEC); Mesenchymal stem cells (MSCs); Human cell line-based β-cells (EndoC-βH3); Mouse insulinoma β cells (β-TC3 cells); Rat heart microvessel endothelial cells (RHMVECs); Primary rat islets microcapillary endothelial cells (IMEC); Bandeiraea simplicifolia (BS-1); von Willebrand factor (vWF).

### 4.1 Formation of pseudoislets from β cell lines and ECs

The co-culture system is an efficient way to study the interaction between islet cells and endothelium. Pseudoislets, formed by the aggregation of β cells, are used *in vitro* to study β cells in a 3D structure and can be employed to investigate the interactions between β cells and ECs. Over the years, various studies have adopted diverse approaches to generate and utilize these pseudoislets (Relevant studies are summarized in Table 3).

ECs can significantly enhance the functionality of pseudoislets and insulin secretion. In 2011, Penko et al. first described mosaic pseudoislets, created *in vitro* by aggregating rat pancreatic cells with rat endothelial progenitor cells. These pseudoislets exhibited improved pancreatic function and insulin secretion compared to traditional methods (Penko et al., 2011). In 2020, Urbanczyk et al. created two-layered pseudoislets composed of human cell line-based β-cells (EndoC-βH3) and HUVECs. A well-defined spatial distribution of the pseudoislets was achieved by controlling the aggregation process using magnetic levitation. The pseudoislets exhibited higher insulin secretory function and a greater number of CD31^+^ HUVECs compared to those formed by spontaneous aggregation (Urbanczyk et al., 2020). In 2023, Porter et al. constructed pseudoislet spheroids coculturing the non-proliferative EndoC-βH5 human β cell line with HUVECs. The 1:3 β cell to HUVEC coculture spheroids displayed higher insulin release (Porter et al., 2023). In 2024, Wang et al. constructed 3D pseudoislets by co-culturing of MIN6 cells and HUVECs in a 2:1 ratio. Functional experiments conducted after 3 days of culture showed increased insulin secretion (Wang et al., 2024a).

ECs also provide protective effects on pseudoislets against stress conditions. In 2021, Wieland et al. cocultured mouse α cells, mouse β cells, and HUVECs in a 1:9:5 ratio to form pseudoislets. After 5 days in culture, oxidative stress was induced using H2O2. The presence of HUVECs significantly reduced oxidative stress in both α and β cells, suggesting a protective effect of ECs on islets (Wieland et al., 2021). Amin et al. (2021) demonstrated that immortalized HUVECs (EA.hy926), when pretreated with the Nrf2 activator rosolic acid (RA) and cocultured with ER-stressed MIN6 cells, exhibited increased Nrf2 levels and reduced ER stress in MIN6 cells. This indicates that RA-sensitized ECs provide protection to β cells (Amin et al., 2021).

ECs have been found to regulate gene expression and signaling pathways of co-cultured islets. Spelios et al. (2018) found that co-cultured pseudoislets enhanced the expression of genes and proteins related to the GLP-1 pathway, partially explaining the improved glucose sensitivity (Spelios, 2018). Daniel et al. (2021) demonstrated that conditioned medium from rat islet microcapillary EC (IMEC) primary cultures could attenuate the first and second phase glucose stimulated insulin release (GSIS) in freshly isolated rat islets and the INS-1E insulinoma cell line. They identified the enzyme triosephosphate isomerase (TPI) as an attenuating factor in the endothelial conditioned medium that promotes insulin secretion via the TPI-sulfonylurea receptor-KATP channel (SUR1-Kir6.2) complex (Daniel et al., 2021). Wang et al. (2024a) demonstrated MIN6 cells cocultured with HUVECs upregulated the expression of E-cadherin, Connexin36, Pdx1 and Mafa, and promoted the secretion of betacellulin (BTC) by MIN6 cells, activating the EGFR-mediated JAK-STAT pathway to enhance MIN6 function (Wang et al., 2024b).

Reciprocally, islet cells were reported to enhance the growth and migration of co-cultured ECs. In 2022, Jung et al. cocultured β-TC6 cells and pancreatic islet endothelial MS1 cells, and found that RNA binding protein HuD in β-TC6 cells promoted the proliferation and migratory behavior of ECs by regulating the expression of Endostatin and Serpin E1 (Jung et al., 2022).

### 4.2 Induction of pancreatic β cells from hPSCs in the presence of ECs

ECs co-culture has been shown to improve the generation of pancreatic β cells derived from hPSCs (Relevant studies are summarized in Table 3). In 2011 and 2016, Talavera-Adame et al. utilized an *in vitro* self-coagulation culture system to generate functional pancreatic β cells from embryoid bodies (EBs) derived from hPSCs, in the presence of human microvascular ECs (HMECs). Compared to controls, EBs cocultured with ECs exhibited higher expression of mature β cell markers and improved insulin secretion *in vitro* (Talavera-Adame et al., 2016; Talavera-Adame et al., 2011). Transplantation of these EBs into diabetic mice resulted in increased C-peptide secretion and significantly lowered blood glucose levels (Talavera-Adame et al., 2016). BMP pathway was activated at the EB-EC interface. EC effects in coculture were mimicked by BMP-2 and inhibited by NOGGIN, indicating BMP pathway activation is central to this process (Talavera-Adame et al., 2016; Talavera-Adame et al., 2011). These findings highlight the beneficial effect of coculture with ECs in enhancing the differentiation and function of hPSCs derived β cells.

The presence of ECs can promote the formation of functional islet cell aggregates. With the continuous improvement of β cell differentiation technologies using hPSCs, Candiello (2018) and Augsornworawat (2019) developed a strategy for assembling hPSCs-derived pancreatic cell aggregates with ECs on hydrogel platforms. The heterogeneous islet organoids expressed β cell and other endocrine markers, demonstrated functionality, and were able to secrete C-peptide and functional insulin in response to glucose stimulation (Augsornworawat et al., 2019; Candiello et al., 2018).

ECs have been reported to regulate β cell fate decisions. In 2022, Chmielowiec et al. cocultured hESC-derived β cells with human primary mesenchymal cells and ECs derived from fetal pancreatic tissues between week 9 and 20 of gestation. They identified week 17 and week 20 as critical time points in the microenvironment that favors β cell differentiation. They found that both ECM and soluble factors secreted by mesenchymal and ECs play a pivotal role in promoting β cell fate decisions. Among these factors, MSCs and ECs express WNT5A, which cooperates with GREMLIN1 to inhibit the BMP pathway during β cell maturation, and endothelial-derived Endocan was shown to promote the differentiation of SST^+^ δ-cells (Chmielowiec et al., 2022).

The combination of macrophages and ECs can efficiently promote maturation of β cells efficiently. In 2024, Yang et al. developed vascularized macrophage-islet (VMI) organoids from hPSCs. They differentiated MEL-1INS/GFP human embryonic stem cells (hESCs) into pancreatic endocrine cells, H9 hESCs into macrophages, and H1 hESCs into ECs by overexpressing ETV2. The VMI organoids exhibited enhanced insulin secretion and calcium mobilization upon high glucose stimulation, indicating the β cells within VMI organoids are more mature than those cultured separately (Yang et al., 2024).

Overall, hPSC-derived β cells offer significant promise for future diabetes therapies, with ECs playing a crucial role in enhancing the development and functional maturation of these cells.

### 4.3 ECs assist in establishing pancreatic organoids from adult stem cells

With the discovery of adult stem cells in various tissues, attempts have been made to utilize pancreatic adult stem cells to generate functional pancreatic organoids *in vitro* (Relevant studies are summarized in Table 3). In 2009, Song et al. isolated ECs from the rat thoracic aorta and cocultured them with freshly isolated rat islets. They found ECs enhanced islet survival and function *in vitro* within 7 days (Song et al., 2009). Similarly, Lehmann et al. (2019) developed a coculture system with mouse islets and cultured pancreatic blood vessels. They first developed a method to isolate and culture pancreatic blood vessels, which could maintain endothelial markers *in vitro*. In the coculture system, the vessels frequently encapsulated the islets. The cocultured islets remained viable, expressed insulin for up to 8 days, and exhibited slight outgrowth (Lehmann et al., 2019). However, both studies only focused on short-term *in vitro* culture and did not involve *in vivo* transplantation.

In 2020, Wang et al. identified a population of stem/progenitor cells characterized by the surface expression of protein C receptor (Procr) within the pancreatic islets of adult mice. This study further developed a methodology for expanding these progenitors and differentiating them into functional islet organoids through coculture with freshly isolated ECs. The resultant islet organoids contained an abundance of β-like cells, along with smaller numbers of α-, δ- and PP-like cells, mirroring the cellular composition of mouse islets. The ECs used for the coculture were isolated from various mouse tissues, such as inguinal fat pads or the skin (Wang et al., 2020; Wang et al., 2022; Misra and Nostro, 2020). This study not only identified islet progenitor cells in the adult mouse pancreas but also achieved their long-term expansion and maturation into functional islet organoids *in vitro*. Importantly, it provides theoretical and technical support for the *in vitro* generation of large quantities of functional pancreatic β cells from islet progenitor cells, opening new avenues for diabetes treatment.

### 4.4 Development of engineered models for coculture of EC and islet cells

With significant advancements in tissue engineering, researchers are now developing sophisticated engineered models to coculture ECs and islet cells, aiming to enhance the functionality and integration of islet transplants (Relevant studies are summarized in Table 3).

Using ECs to create a biocompatible scaffold enhanced the engraftment of islets. Everwien et al. (2020) created a decellularized, re-endothelialized, and endocrine-repopulated rat liver, referred to as the Neo-Pancreas. In their study, ECs formed a monolayer during cultivation. After 3 days of cultivation, the Neo-Pancreas exhibited increased insulin secretion in response to high glucose stimulation (Everwien et al., 2020).

Thorough vascular penetration is required with EC-lined scaffolds to sustain islet function. In 2020, Rambøl successfully developed an *in vitro* microfluidic platform to study the interactions between islets and the microvascular network. By utilizing MSCs and HUVECs, a perfusable microvascular network was constructed. *In vitro* studies revealed that rat islets could locally recruit microvessels; however, within 5 days, these microvessels enveloped only the islet surface without penetrating the interior (Rambol et al., 2020). In the same year, Seo et al. used a cell-laden collagen sheet to coculture MIN6 and MS1 cells, forming blood vessel-like structures and islet-like clusters. These cells maintained over 85% viability within 2 weeks and exhibited higher levels of insulin secretion (Seo et al., 2020). Wassmer et al. (2021) generated and transplanted pre-vascularized insulin-secreting organoids composed of rat primary islet cells, HUVECs, and hAECs. The pre-vascularized islet organoids exhibited enhanced function, better engraftment and improved vascularization (Wassmer et al., 2021).

ECs have been shown to promote the expression of relevant basement membrane proteins, which support β cells survival and serve as a primary source of ECM proteins. These proteins facilitate cell engraftment and the overall stability and function of the engineered tissue (Zbinden et al., 2021).

Organs-on-chips meticulously incorporate cells, tissue-to-tissue interfaces, fluidic dynamics, and other biomimetic microenvironments, thereby faithfully replicating organ-specific functions (Tao et al., 2019) and inter-organ interactions (Tao et al., 2022). The vascularization process within the chip significantly enhanced the growth, maturation, and functional performance of the islet organoids. Hospodiuk-Karwowski et al. (2022) developed a hydrogel-based perfusable, vascularized pancreas-on-a-chip device using rat heart microvessel ECs and mouse insulinoma beta (β)TC3 cells (Hospodiuk-Karwowski et al., 2022). In 2024, Quintard et al. developed a microfluidic platform to establish and monitor the formation of endothelial networks around islet spheroids, which were cultured on-chip for up to 30 days. The islet organoid growth, maturation, and function were enhanced when vascularized on-chip (Quintard et al., 2024).

The aim of co-culturing ECs and β cells in organoid systems is to improve β cell differentiation and maturity, more accurately study the islet microenvironment, and enhance vascularization in mouse organoids and human islets prior to transplantation (Beydag-Tasöz et al., 2023). Overall, these engineered models present promising approaches for developing functional and stably vascularized islets that could potentially improve current clinical shortcomings in islet transplantation. These findings highlight ECs as an important factor supporting the generation and maintenance of functional pancreatic islet organoids in culture.

### 4.5 Regional EC heterogeneity

ECs exhibit significant phenotypic heterogeneity across different tissues, reflected in their morphology, molecular markers, and functional adaptations to the microenvironment. This heterogeneity is also observed in ECs in the pancreatic vasculature (Chen et al., 2021; Khan et al., 2024). Specifically, intra-islet ECs differ markedly from exocrine ECs in their physiological and functional roles. In mice, intra-islet ECs create a unique microenvironment by expressing specific markers (CD31^+^ Emcn^hi^) and secreting higher level of paracrine factors (like Hgf and Igf1/2), which support the development, function, and survival of β cells (Chen et al., 2021). Pancreatic ECs exhibit high permeability and specialized intercellular junctions, facilitating efficient nutrient and hormone transport essential for β cell metabolic needs and insulin secretion (Khan et al., 2024).

Coculturing hESC-derived pancreatic progenitors with primary mesenchymal cells and ECs increased C-peptide^+^ cells proportion compared to coculture with HUVECs and MEFs. Urbanczyk et al. demonstrated that co-culturing isolated human islets with human pancreatic microvascular ECs significantly improved islet functionality compared to co-culture with HUVECs (Urbanczyk et al., 2024). A study comparing EC from different portions of pancreas also found mesenchymal cells and ECs from the head of the pancreas exhibited a seemingly more superior supportive capacity and could induce more C-peptide^+^ cells (Chmielowiec et al., 2022). Besides, Aghazadeh, (2021) showed that ready-made microvessels were more effective than single HUVEC in connecting with the host vasculature, ensuring successful islet transplantation (Aghazadeh et al., 2021).

In conclusion, co-culture with organ-specific and primary ECs showed improved organoid function and islet transplantation outcome. Future research should focus on deciphering the molecular cues ECs exert, in order to benefit clinical transplantation.

## 5 Discussion

In 2000, Shapiro’s team pioneered a new era of islet transplantation for the treatment of T1DM, freeing all seven recipients from exogenous insulin (Shapiro et al., 2000). Still, challenges remain to be resolved including donor shortage, graft survival, immune rejection, and long-term function maintenance.

To address the donor shortage issue, many groups developed differentiation protocols to generate stem cell derived islets. For example, in the VX-880 clinical trial conducted by Vertex, 11 out of 12 patients transplanted with ESC-derived islets discontinued insulin use after a period of time (Vertex, 2024; Medicine, 2025). In another study, researchers used chemically induced stem cell-derived islets to restore glucose regulation in a T1DM patient, and resulted in complete insulin independence starting from day 78 after transplantation (Wang et al., 2024c). Despite the recent advancement in cell therapy, it seems that up to 3 months are required for the graft to reach optimal function (devoid of exogenous insulin use). Hence, whether the use of EC co-culture could accelerate the functional maturation of transplanted grafts is definitely worth investigating. To move forward, considerations on EC source, purity, phenotype, scalability, mode of administration and respective quality assessments should be addressed to ensure their safety and efficacy in clinical applications.

Conventional transplantation through the portal vein is both invasive and difficult to monitor. Subcutaneous transplantation has been an attractive alternative for islet transplantation, due to its simplicity of surgical procedures, minimal invasiveness, capacity to accommodate relatively large graft volumes, ease of monitoring, removal or replacement. For this purpose, various subcutaneous vascularization devices are developed in order to protect graft from immune attack while still able to nourish through circulation. But scalability has always been a challenge for such devices. Recent advances in biomaterials and bioengineering have enabled successful pre-vascularization of subcutaneous sites and the development of immunosuppression-free strategies (Wang et al., 2023). The SHEATH system has explored the potential for scalability using a large animal (minipig) model and the recently launched clinical trial (NCT05073302) on pre-vascularization technology, which will provide valuable insights to guide further optimization and clinical translation of the SHEATH system. Besides subcutaneous transplantation, recent studies from Deng’s lab have proposed abdominal anterior rectus sheath as an alternative transplantation site for its abundant vasculature (Wang et al., 2024d; Liang et al., 2023). Whether combining a pre-vascularization device will yield better results awaits further investigation. Ultimately, the scalability of cell replacement therapy is crucial for achieving clinical benefits, and requires interdisciplinary collaboration and technological innovation.

This review summarizes the recent studies on the role of vascular ECs in islet development, transplantation, and organoid culture. Continued exploration of these areas holds promise for enhancing the efficacy of islet transplantation and is undoubtedly useful for developing novel therapeutic strategies.

## References

[B1] Aghazadeh Y. Poon F. Sarangi F. Wong F. T. M. Khan S. T. Sun X. (2021). Microvessels support engraftment and functionality of human islets and hESC-derived pancreatic progenitors in diabetes models. Cell Stem Cell 28 (11), 1936–1949 e8. 10.1016/j.stem.2021.08.001 34480863

[B2] Al-Hasani K. Marikar S. N. Kaipananickal H. Maxwell S. Okabe J. Khurana I. (2024). EZH2 inhibitors promote beta-like cell regeneration in young and adult type 1 diabetes donors. Signal Transduct. Target Ther. 9 (1), 2. 10.1038/s41392-023-01707-x 38161208 PMC10757994

[B3] Amin K. N. Palanisamy R. Sarada D. V. L. Ali D. Suzuki T. Ramkumar K. M. (2021). Effect of Rosolic acid on endothelial dysfunction under ER stress in pancreatic microenvironment. Free Radic. Res. 55 (6), 698–713. 10.1080/10715762.2021.1892090 33788639

[B4] An D. Chiu A. Flanders J. A. Song W. Shou D. Lu Y. C. (2018). Designing a retrievable and scalable cell encapsulation device for potential treatment of type 1 diabetes. Proc. Natl. Acad. Sci. U. S. A. 115 (2), E263-E272–E272. 10.1073/pnas.1708806115 29279393 PMC5777032

[B5] Andrew R Pepper R. P. Gala-Lopez B. MacGillivary A. Mazzuca D. M. White D. J. G. Toleikis P. M. (2015). Diabetes is reversed in a murine model by marginal mass syngeneic islet transplantation using a subcutaneous cell pouch device. Transplantation 99, 2294–2300. 10.1097/TP.0000000000000864 26308506 PMC4623852

[B6] Augsornworawat P. Velazco-Cruz L. Song J. Millman J. R. (2019). A hydrogel platform for *in vitro* three dimensional assembly of human stem cell-derived islet cells and endothelial cells. Acta Biomater. 97, 272–280. 10.1016/j.actbio.2019.08.031 31446050 PMC6801041

[B7] Barba-Gutierrez D. A. Daneri-Navarro A. Villagomez-Mendez J. J. A. Kanamune J. Robles-Murillo A. K. Sanchez-Enriquez S. (2016). Facilitated engraftment of isolated islets coated with expanded vascular endothelial cells for islet transplantation. Transpl. Proc. 48 (2), 669–672. 10.1016/j.transproceed.2016.02.036 27110026

[B8] Baudin B. Bruneel A. Bosselut N. Vaubourdolle M. (2007). A protocol for isolation and culture of human umbilical vein endothelial cells. Nat. Protoc. 2 (3), 481–485. 10.1038/nprot.2007.54 17406610

[B9] Bellin M. D. Dunn T. B. (2020). Transplant strategies for type 1 diabetes: whole pancreas, islet and porcine beta cell therapies. Diabetologia 63 (10), 2049–2056. 10.1007/s00125-020-05184-7 32894315

[B10] Berclaz C. Szlag D. Nguyen D. Extermann J. Bouwens A. Marchand P. J. (2016). Label-free fast 3D coherent imaging reveals pancreatic islet micro-vascularization and dynamic blood flow. Biomed. Opt. Express 7 (11), 4569–4580. 10.1364/boe.7.004569 27895996 PMC5119596

[B11] Beydag-Tasöz B. S. Yennek S. Grapin-Botton A. (2023). Towards a better understanding of diabetes mellitus using organoid models. Nat. Rev. Endocrinol. 19 (4), 232–248. 10.1038/s41574-022-00797-x 36670309 PMC9857923

[B12] Biarnes M. Montolio M. Nacher V. Raurell M. Soler J. Montanya E. (2002). Beta-cell death and mass in syngeneically transplanted islets exposed to short- and long-term hyperglycemia. Diabetes 51 (1), 66–72. 10.2337/diabetes.51.1.66 11756324

[B13] Bonder C. S. Sun W. Y. Matthews T. Cassano C. Li X. Ramshaw H. S. (2009). Sphingosine kinase regulates the rate of endothelial progenitor cell differentiation. Blood 113 (9), 2108–2117. 10.1182/blood-2008-07-166942 19109558 PMC2651020

[B14] Bonner-Weir S. (2000). Perspective: postnatal pancreatic beta cell growth. Endocrinology 141 (6), 1926–1929. 10.1210/endo.141.6.7567 10830272

[B15] Bowers D. T. Song W. Wang L. H. Ma M. (2019). Engineering the vasculature for islet transplantation. Acta Biomater. 95, 131–151. 10.1016/j.actbio.2019.05.051 31128322 PMC6824722

[B16] Brelje T. C. Scharp D. W. Lacy P. E. Ogren L. Talamantes F. Robertson M. (1993). Effect of homologous placental lactogens, prolactins, and growth hormones on islet B-cell division and insulin secretion in rat, mouse, and human islets: implication for placental lactogen regulation of islet function during pregnancy. Endocrinology 132 (2), 879–887. 10.1210/endo.132.2.8425500 8425500

[B17] Brissova M. Shostak A. Fligner C. L. Revetta F. L. Washington M. K. Powers A. C. (2015). Human islets have fewer blood vessels than mouse islets and the density of islet vascular structures is increased in type 2 diabetes. J. Histochem Cytochem 63 (8), 637–645. 10.1369/0022155415573324 26216139 PMC4530394

[B18] Burganova G. Bridges C. Thorn P. Landsman L. (2021). The role of vascular cells in pancreatic beta-cell function. Front. Endocrinol. (Lausanne) 12, 667170. 10.3389/fendo.2021.667170 33981287 PMC8109179

[B19] Candiello J. Grandhi T. S. P. Goh S. K. Vaidya V. Lemmon-Kishi M. Eliato K. R. (2018). 3D heterogeneous islet organoid generation from human embryonic stem cells using a novel engineered hydrogel platform. Biomaterials 177, 27–39. 10.1016/j.biomaterials.2018.05.031 29883914

[B20] Carlsson P. O. Palm F. Mattsson G. (2002). Low revascularization of experimentally transplanted human pancreatic islets. J. Clin. Endocrinol. Metab. 87 (12), 5418–5423. 10.1210/jc.2002-020728 12466329

[B21] Casamitjana J. Espinet E. Rovira M. (2022). Pancreatic organoids for regenerative medicine and cancer research. Front. Cell Dev. Biol. 10, 886153. 10.3389/fcell.2022.886153 35592251 PMC9110799

[B22] Chen H. Gu X. Liu Y. Wang J. Wirt S. E. Bottino R. (2011). PDGF signalling controls age-dependent proliferation in pancreatic beta-cells. Nature 478 (7369), 349–355. 10.1038/nature10502 21993628 PMC3503246

[B23] Chen J. Lippo L. Labella R. Tan S. L. Marsden B. D. Dustin M. L. (2021). Decreased blood vessel density and endothelial cell subset dynamics during ageing of the endocrine system. EMBO J. 40 (1), e105242. 10.15252/embj.2020105242 33215738 PMC7780152

[B24] Chen Q. D. Liu L. Zhao X. H. Liang J. B. Li S. W. (2023). Challenges and opportunities in the islet transplantation microenvironment: a comprehensive summary of inflammatory cytokine, immune cells, and vascular endothelial cells. Front. Immunol. 14, 1293762. 10.3389/fimmu.2023.1293762 38111575 PMC10725940

[B25] Chmielowiec J. Szlachcic W. J. Yang D. Scavuzzo M. A. Wamble K. Sarrion-Perdigones A. (2022). Human pancreatic microenvironment promotes β-cell differentiation via non-canonical WNT5A/JNK and BMP signaling. Nat. Commun. 13 (1), 1952. 10.1038/s41467-022-29646-1 35414140 PMC9005503

[B26] Cleaver O. Dor Y. (2012). Vascular instruction of pancreas development. Development 139 (16), 2833–2843. 10.1242/dev.065953 22833471 PMC3403096

[B27] Craig R. Halberstadt D. W. Emerich D. Goddard M. Vasconcellos A. V. Curry W. (2005). Subcutaneous transplantation of islets into streptozocin-induced diabetic rats. Cell Transplant. 14, 595–605. 10.3727/000000005783982792 16355568

[B28] Crawford L. A. Guney M. A. Oh Y. A. Deyoung R. A. Valenzuela D. M. Murphy A. J. (2009). Connective tissue growth factor (CTGF) inactivation leads to defects in islet cell lineage allocation and beta-cell proliferation during embryogenesis. Mol. Endocrinol. 23 (3), 324–336. 10.1210/me.2008-0045 19131512 PMC2654514

[B29] Crawford S. E. Stellmach V. Murphy-Ullrich J. E. Ribeiro S. M. Lawler J. Hynes R. O. (1998). Thrombospondin-1 is a major activator of TGF-beta1 *in vivo* . Cell 93 (7), 1159–1170. 10.1016/s0092-8674(00)81460-9 9657149

[B30] Critser P. J. Yoder M. C. (2010). Endothelial colony-forming cell role in neoangiogenesis and tissue repair. Curr. Opin. Organ Transpl. 15 (1), 68–72. 10.1097/MOT.0b013e32833454b5 PMC2880951 19898235

[B31] Cunha D. A. Cito M. Carlsson P. O. Vanderwinden J. M. Molkentin J. D. Bugliani M. (2016). Thrombospondin 1 protects pancreatic β-cells from lipotoxicity via the PERK-NRF2 pathway. Cell Death Differ. 23 (12), 1995–2006. 10.1038/cdd.2016.89 27588705 PMC5136495

[B32] Cunha D. A. Cito M. Grieco F. A. Cosentino C. Danilova T. Ladrière L. (2017). Pancreatic β-cell protection from inflammatory stress by the endoplasmic reticulum proteins thrombospondin 1 and mesencephalic astrocyte-derived neutrotrophic factor (MANF). J. Biol. Chem. 292 (36), 14977–14988. 10.1074/jbc.M116.769877 28698383 PMC5592674

[B33] Daniel B. Livne A. Cohen G. Kahremany S. Sasson S. (2021). Endothelial cell-derived triosephosphate isomerase attenuates insulin secretion from pancreatic beta cells of male rats. Endocrinology 162 (3), bqaa234. 10.1210/endocr/bqaa234 33341896

[B34] Davalli A. M. Ogawa Y. Ricordi C. Scharp D. W. Bonner-Weir S. Weir G. C. (1995). A selective decrease in the beta cell mass of human islets transplanted into diabetic nude mice. Transplantation 59 (6), 817–820. 10.1097/00007890-199503000-00003 7701574

[B35] David S. R. Lai P. P. N. Chellian J. Chakravarthi S. Rajabalaya R. (2023). Influence of rutin and its combination with metformin on vascular functions in type 1 diabetes. Sci. Rep. 13 (1), 12423. 10.1038/s41598-023-39442-6 37528147 PMC10394083

[B36] Dawson D. W. Pearce S. F. Zhong R. Silverstein R. L. Frazier W. A. Bouck N. P. (1997). CD36 mediates the *in vitro* inhibitory effects of thrombospondin-1 on endothelial cells. J. Cell Biol. 138 (3), 707–717. 10.1083/jcb.138.3.707 9245797 PMC2141641

[B37] De Falco E. Porcelli D. Torella A. R. Straino S. Iachininoto M. G. Orlandi A. (2004). SDF-1 involvement in endothelial phenotype and ischemia-induced recruitment of bone marrow progenitor cells. Blood 104 (12), 3472–3482. 10.1182/blood-2003-12-4423 15284120

[B38] de Klerk E. Hebrok M. (2021). Stem cell-based clinical trials for diabetes mellitus. Front. Endocrinol. (Lausanne) 12, 631463. 10.3389/fendo.2021.631463 33716982 PMC7953062

[B39] Dobson K. R. Reading L. Haberey M. Marine X. Scutt A. (1999). Centrifugal isolation of bone marrow from bone: an improved method for the recovery and quantitation of bone marrow osteoprogenitor cells from rat tibiae and femurae. Calcif. Tissue Int. 65 (5), 411–413. 10.1007/s002239900723 10541770

[B40] Drott C. J. Olerud J. Emanuelsson H. Christoffersson G. Carlsson P. O. (2012). Sustained beta-cell dysfunction but normalized islet mass in aged thrombospondin-1 deficient mice. PLoS One 7 (10), e47451. 10.1371/journal.pone.0047451 23094049 PMC3477147

[B41] Erdem N. Chen K. T. Qi M. Zhao Y. Wu X. Garcia I. (2023). Thrombospondin-1, CD47, and SIRPα display cell-specific molecular signatures in human islets and pancreata. Am. J. Physiol. Endocrinol. Metab. 324 (4), E347–e357. 10.1152/ajpendo.00221.2022 36791324 PMC11967708

[B42] Eriksson O. Eich T. Sundin A. Tibell A. Tufveson G. Andersson H. (2009). Positron emission tomography in clinical islet transplantation. Am. J. Transpl. 9 (12), 2816–2824. 10.1111/j.1600-6143.2009.02844.x 19845588

[B43] Everwien H. Keshi E. Hillebrandt K. H. Ludwig B. Weinhart M. Tang P. (2020). Engineering an endothelialized, endocrine Neo-Pancreas: evaluation of islet functionality in an *ex vivo* model. Acta Biomater. 117, 213–225. 10.1016/j.actbio.2020.09.022 32949822

[B44] Ferrara N. Davis-Smyth T. (1997). The biology of vascular endothelial growth factor. Endocr. Rev. 18 (1), 4–25. 10.1210/edrv.18.1.0287 9034784

[B45] Frueh F. S. Später T. Lindenblatt N. Calcagni M. Giovanoli P. Scheuer C. (2017b). Adipose tissue-derived microvascular fragments improve vascularization, lymphangiogenesis, and integration of dermal skin substitutes. J. Invest Dermatol 137 (1), 217–227. 10.1016/j.jid.2016.08.010 27574793

[B46] Frueh F. S. Später T. Scheuer C. Menger M. D. Laschke M. W. (2017a). Isolation of murine adipose tissue-derived microvascular fragments as vascularization units for tissue engineering. J. Vis. Exp. 122, 55721. 10.3791/55721 PMC5565147 28518106

[B47] Garikipati V. N. S. Kishore R. (2017). Endothelial progenitor cells: procedure for cell isolation and applications. Methods Mol. Biol. 1553, 85–89. 10.1007/978-1-4939-6756-8_7 28229409

[B48] Gebe J. A. Preisinger A. Gooden M. D. D'Amico L. A. Vernon R. B. (2018). Local, controlled release *in vivo* of vascular endothelial growth factor within a subcutaneous scaffolded islet implant reduces early islet necrosis and improves performance of the graft. Cell Transpl. 27 (3), 531–541. 10.1177/0963689718754562 PMC6038045 29756517

[B49] Gittes G. K. (2009). Developmental biology of the pancreas: a comprehensive review. Dev. Biol. 326 (1), 4–35. 10.1016/j.ydbio.2008.10.024 19013144

[B50] Golocheikine A. Tiriveedhi V. Angaswamy N. Benshoff N. Sabarinathan R. Mohanakumar T. (2010). Cooperative signaling for angiogenesis and neovascularization by VEGF and HGF following islet transplantation. Transplantation 90 (7), 725–731. 10.1097/TP.0b013e3181ef8a63 20714284

[B51] Guney M. A. Petersen C. P. Boustani A. Duncan M. R. Gunasekaran U. Menon R. (2011). Connective tissue growth factor acts within both endothelial cells and beta cells to promote proliferation of developing beta cells. Proc. Natl. Acad. Sci. U. S. A. 108 (37), 15242–15247. 10.1073/pnas.1100072108 21876171 PMC3174622

[B52] Henderson J. R. Moss M. C. (1985). A morphometric study of the endocrine and exocrine capillaries of the pancreas. Q. J. Exp. Physiol. 70 (3), 347–356. 10.1113/expphysiol.1985.sp002920 3898188

[B53] Henry B. M. Skinningsrud B. Saganiak K. Pękala P. A. Walocha J. A. Tomaszewski K. A. (2019). Development of the human pancreas and its vasculature - an integrated review covering anatomical, embryological, histological, and molecular aspects. Ann. Anat. 221, 115–124. 10.1016/j.aanat.2018.09.008 30300687

[B54] Hoesli C. A. Kieffer T. J. (2021). Pancreatic islets in bed with microvasculature-companions for life. Cell Rep. Med. 2 (11), 100454. 10.1016/j.xcrm.2021.100454 34841297 PMC8607006

[B55] Hogan M. F. Hull R. L. (2017). The islet endothelial cell: a novel contributor to beta cell secretory dysfunction in diabetes. Diabetologia 60 (6), 952–959. 10.1007/s00125-017-4272-9 28396983 PMC5505567

[B56] Hogrebe N. J. Ishahak M. Millman J. R. (2023). Developments in stem cell-derived islet replacement therapy for treating type 1 diabetes. Cell Stem Cell 30 (5), 530–548. 10.1016/j.stem.2023.04.002 37146579 PMC10167558

[B57] Homma J. Sekine H. Shimizu T. (2023). Tricultured cell sheets develop into functional pancreatic islet tissue with a vascular network. Tissue Eng. Part A 29 (7-8), 211–224. 10.1089/ten.TEA.2022.0167 36565034

[B58] Hospodiuk-Karwowski M. Chi K. Pritchard J. Catchmark J. M. (2022). Vascularized pancreas-on-a-chip device produced using a printable simulated extracellular matrix. Biomed. Mater 17 (6), 065006. 10.1088/1748-605X/ac8c74 36001993

[B59] Hur J. Yoon C. H. Kim H. S. Choi J. H. Kang H. J. Hwang K. K. (2004). Characterization of two types of endothelial progenitor cells and their different contributions to neovasculogenesis. Arterioscler. Thromb. Vasc. Biol. 24 (2), 288–293. 10.1161/01.ATV.0000114236.77009.06 14699017

[B60] Ivkovic S. Yoon B. S. Popoff S. N. Safadi F. F. Libuda D. E. Stephenson R. C. (2003). Connective tissue growth factor coordinates chondrogenesis and angiogenesis during skeletal development. Development 130 (12), 2779–2791. 10.1242/dev.00505 12736220 PMC3360973

[B61] Jacquemin P. Yoshitomi H. Kashima Y. Rousseau G. G. Lemaigre F. P. Zaret K. S. (2006). An endothelial-mesenchymal relay pathway regulates early phases of pancreas development. Dev. Biol. 290 (1), 189–199. 10.1016/j.ydbio.2005.11.023 16386727

[B62] James D. Nam H. s. Seandel M. Nolan D. Janovitz T. Tomishima M. (2010). Expansion and maintenance of human embryonic stem cell-derived endothelial cells by TGFbeta inhibition is Id1 dependent. Nat. Biotechnol. 28 (2), 161–166. 10.1038/nbt.1605 20081865 PMC2931334

[B63] Jennings R. E. Berry A. A. Strutt J. P. Gerrard D. T. Hanley N. A. (2015). Human pancreas development. Development 142 (18), 3126–3137. 10.1242/dev.120063 26395141

[B183] Jennings R. E. Berry A. A. Kirkwood-Wilson R. Roberts N. A. Hearn T. Salisbury R. J. (2013). Development of the human pancreas from foregut to endocrine commitment. Diabetes 62 (10), 3514–3522. 10.2337/db12-1479 23630303 PMC3781486

[B64] Jiang L. Shen Y. Liu Y. Zhang L. Jiang W. (2022). Making human pancreatic islet organoids: progresses on the cell origins, biomaterials and three-dimensional technologies. Theranostics 12 (4), 1537–1556. 10.7150/thno.66670 35198056 PMC8825586

[B65] Jimenez B. Volpert O. V. Crawford S. E. Febbraio M. Silverstein R. L. Bouck N. (2000). Signals leading to apoptosis-dependent inhibition of neovascularization by thrombospondin-1. Nat. Med. 6 (1), 41–48. 10.1038/71517 10613822

[B66] Johansson M. Andersson A. Carlsson P. O. Jansson L. (2006a). Perinatal development of the pancreatic islet microvasculature in rats. J. Anat. 208 (2), 191–196. 10.1111/j.1469-7580.2006.00520.x 16441563 PMC2100194

[B67] Johansson M. Mattsson G. Andersson A. Jansson L. Carlsson P. O. (2006b). Islet endothelial cells and pancreatic beta-cell proliferation: studies *in vitro* and during pregnancy in adult rats. Endocrinology 147 (5), 2315–2324. 10.1210/en.2005-0997 16439446

[B68] Johansson U. Rasmusson I. Niclou S. P. Forslund N. Gustavsson L. Nilsson B. (2008). Formation of composite endothelial cell-mesenchymal stem cell islets: a novel approach to promote islet revascularization. Diabetes 57 (9), 2393–2401. 10.2337/db07-0981 18519803 PMC2518490

[B69] Jung M. Ryu S. Kim C. Cha S. Kang H. Ji E. (2022). RNA binding protein HuD mediates the crosstalk between β cells and islet endothelial cells by the regulation of Endostatin and Serpin E1 expression. Cell Death Dis. 13 (12), 1019. 10.1038/s41419-022-05465-6 36470872 PMC9722926

[B70] Kaido T. Yebra M. Cirulli V. Montgomery A. M. (2004). Regulation of human beta-cell adhesion, motility, and insulin secretion by collagen IV and its receptor alpha1beta1. J. Biol. Chem. 279 (51), 53762–53769. 10.1074/jbc.M411202200 15485856

[B71] Kang S. Park H. S. Jo A. Hong S. H. Lee H. N. Lee Y. Y. (2012). Endothelial progenitor cell cotransplantation enhances islet engraftment by rapid revascularization. Diabetes 61 (4), 866–876. 10.2337/db10-1492 22362173 PMC3314353

[B72] Karanth S. S. Sun S. Bi H. Ye K. Jin S. (2021). Angiopoietins stimulate pancreatic islet development from stem cells. Sci. Rep. 11 (1), 13558. 10.1038/s41598-021-92922-5 34193893 PMC8245566

[B73] Katsumoto K. Kume S. (2011). Endoderm and mesoderm reciprocal signaling mediated by CXCL12 and CXCR4 regulates the migration of angioblasts and establishes the pancreatic fate. Development 138 (10), 1947–1955. 10.1242/dev.058719 21490062

[B74] Kawaguchi Y. Cooper B. Gannon M. Ray M. MacDonald R. J. Wright C. V. E. (2002). The role of the transcriptional regulator Ptf1a in converting intestinal to pancreatic progenitors. Nat. Genet. 32 (1), 128–134. 10.1038/ng959 12185368

[B75] Kawakami Y. Iwata H. Gu Y. Miyamoto M. Murakami Y. Yamasaki T. (2000). Modified subcutaneous tissue with neovascularization is useful as the site for pancreatic islet transplantation. Cell Transpl. 9 (5), 729–732. 10.1177/096368970000900523 11144974

[B76] Kawakami Y. Iwata H. Gu Y. J. Miyamoto M. Murakami Y. Balamurugan A. N. (2001). Successful subcutaneous pancreatic islet transplantation using an angiogenic growth factor-releasing device. Pancreas 23 (4), 375–381. 10.1097/00006676-200111000-00007 11668206

[B77] Khan S. T. Ahuja N. Taïb S. Vohra S. Cleaver O. Nunes S. S. (2024). Single-cell meta-analysis uncovers the pancreatic endothelial cell transcriptomic signature and reveals a key role for NKX2-3 in PLVAP expression. Arterioscler. Thromb. Vasc. Biol. 44, 2596–2615. 10.1161/atvbaha.124.321781 39445426 PMC11594071

[B78] Kobayashi N. (2008). The current status of islet transplantation and its perspectives. Rev. Diabet. Stud. 5 (3), 136–143. 10.1900/RDS.2008.5.136 19099085 PMC2613274

[B79] Kragl M. Lammert E. (2010). Basement membrane in pancreatic islet function. Adv. Exp. Med. Biol. 654, 217–234. 10.1007/978-90-481-3271-3_10 20217500

[B80] Krenning G. van Luyn M. J. Harmsen M. C. (2009). Endothelial progenitor cell-based neovascularization: implications for therapy. Trends Mol. Med. 15 (4), 180–189. 10.1016/j.molmed.2009.02.001 19303359

[B81] Kushner J. A. (2013). The role of aging upon beta cell turnover. J. Clin. Invest 123 (3), 990–995. 10.1172/JCI64095 23454762 PMC3582123

[B82] Lacy P. E. Hegre O. D. Gerasimidi-Vazeou A. Gentile F. T. Dionne K. E. (1991). Maintenance of normoglycemia in diabetic mice by subcutaneous xenografts of encapsulated islets. Science 254 (5039), 1782–1784. 10.1126/science.1763328 1763328

[B83] Lai Y. Schneider D. Kidszun A. Hauck-Schmalenberger I. Breier G. Brandhorst D. (2005). Vascular endothelial growth factor increases functional beta-cell mass by improvement of angiogenesis of isolated human and murine pancreatic islets. Transplantation 79 (11), 1530–1536. 10.1097/01.tp.0000163506.40189.65 15940042

[B84] Lammert E. Cleaver O. Melton D. (2001). Induction of pancreatic differentiation by signals from blood vessels. Science 294 (5542), 564–567. 10.1126/science.1064344 11577200

[B85] Laschke M. W. Menger M. D. (2015). Adipose tissue-derived microvascular fragments: natural vascularization units for regenerative medicine. Trends Biotechnol. 33 (8), 442–448. 10.1016/j.tibtech.2015.06.001 26137863

[B86] Laschke M. W. Menger M. D. (2016). Prevascularization in tissue engineering: current concepts and future directions. Biotechnol. Adv. 34 (2), 112–121. 10.1016/j.biotechadv.2015.12.004 26674312

[B87] Laschke M. W. Menger M. D. (2022). The simpler, the better: tissue vascularization using the body's own resources. Trends Biotechnol. 40 (3), 281–290. 10.1016/j.tibtech.2021.07.002 34404555

[B88] Lehmann V. Andersen P. L. Damodaran R. G. Vermette P. (2019). Method for isolation of pancreatic blood vessels, their culture and coculture with islets of langerhans. Biotechnol. Prog. 35 (2), e2745. 10.1002/btpr.2745 30421867

[B89] Li W. Nakanishi M. Zumsteg A. Shear M. Wright C. Melton D. A. (2014). *In vivo* reprogramming of pancreatic acinar cells to three islet endocrine subtypes. Elife 3, e01846. 10.7554/eLife.01846 24714494 PMC3977343

[B90] Liang Z. Sun D. Lu S. Lei Z. Wang S. Luo Z. (2023). Implantation underneath the abdominal anterior rectus sheath enables effective and functional engraftment of stem-cell-derived islets. Nat. Metab. 5 (1), 29–40. 10.1038/s42255-022-00713-7 36624157

[B91] Liu Y. Yang M. Cui Y. Yao Y. Liao M. Yuan H. (2020). A novel prevascularized tissue-engineered chamber as a site for allogeneic and xenogeneic islet transplantation to establish a bioartificial pancreas. PLoS One 15 (12), e0234670. 10.1371/journal.pone.0234670 33270650 PMC7714105

[B92] Luan N. M. Iwata H. (2014). Long-term allogeneic islet graft survival in prevascularized subcutaneous sites without immunosuppressive treatment. Am. J. Transpl. 14 (7), 1533–1542. 10.1111/ajt.12739 24909185

[B93] Lysy P. A. Weir G. C. Bonner-Weir S. (2013). Making beta cells from adult cells within the pancreas. Curr. Diab Rep. 13 (5), 695–703. 10.1007/s11892-013-0400-1 23925431 PMC3798031

[B94] Mahou R. Zhang D. K. Y. Vlahos A. E. Sefton M. V. (2017). Injectable and inherently vascularizing semi-interpenetrating polymer network for delivering cells to the subcutaneous space. Biomaterials 131, 27–35. 10.1016/j.biomaterials.2017.03.032 28371625

[B95] Mateus Gonçalves L. Fahd Qadir M. M. Boulina M. Makhmutova M. Pereira E. Almaça J. (2023). Pericyte dysfunction and impaired vasomotion are hallmarks of islets during the pathogenesis of type 1 diabetes. Cell Rep. 42 (8), 112913. 10.1016/j.celrep.2023.112913 37531253 PMC10529889

[B96] Mathieu C. Martens P. J. Vangoitsenhoven R. (2021). One hundred years of insulin therapy. Nat. Rev. Endocrinol. 17 (12), 715–725. 10.1038/s41574-021-00542-w 34404937

[B97] Medicine (2025). Tolerability, and efficacy study of VX-880 in participants with type 1 diabetes.

[B98] Meier J. J. Butler A. E. Galasso R. Butler P. C. (2006). Hyperinsulinemic hypoglycemia after gastric bypass surgery is not accompanied by islet hyperplasia or increased β-cell turnover. Diabetes Care 29 (7), 1554–1559. 10.2337/dc06-0392 16801578

[B99] Misra P. S. Nostro M. C. (2020). Islet-resident endocrine progenitors: a new hope for beta cell PROCReation? Cell Stem Cell 26 (4), 471–473. 10.1016/j.stem.2020.03.008 32243804

[B100] Nadal A. Quesada I. Soria B. J. T. J. o.P. (1999). Homologous and heterologous asynchronicity between identified alpha-beta- and delta-cells within intact islets of Langerhans in the mouse. J. Physiol. 517 (1), 85–93. 10.1111/j.1469-7793.1999.0085z.x 10226151 PMC2269319

[B101] Nalbach L. Müller D. Wrublewsky S. Metzger W. Menger M. D. Laschke M. W. (2021a). Microvascular fragment spheroids: three-dimensional vascularization units for tissue engineering and regeneration. J. Tissue Eng. 12, 20417314211035593. 10.1177/20417314211035593 34471514 PMC8404660

[B102] Nalbach L. Roma L. P. Schmitt B. M. Becker V. Körbel C. Wrublewsky S. (2021b). Improvement of islet transplantation by the fusion of islet cells with functional blood vessels. EMBO Mol. Med. 13 (1), e12616. 10.15252/emmm.202012616 33135383 PMC7799357

[B103] Naqvi R. A. Naqvi A. (2023). Co-transplantation with mesenchymal stem cells and endothelial cells improvise islet engraftment and survival in STZ treated hyperglycemic mice. bioRxiv, 525444. 10.1101/2023.01.24.525444

[B104] Naziruddin B. Iwahashi S. Kanak M. A. Takita M. Itoh T. Levy M. F. (2014). Evidence for instant blood-mediated inflammatory reaction in clinical autologous islet transplantation. Am. J. Transpl. 14 (2), 428–437. 10.1111/ajt.12558 24447621

[B105] Nikolova G. Jabs N. Konstantinova I. Domogatskaya A. Tryggvason K. Sorokin L. (2006). The vascular basement membrane: a niche for insulin gene expression and Beta cell proliferation. Dev. Cell 10 (3), 397–405. 10.1016/j.devcel.2006.01.015 16516842

[B106] Oh B. J. Oh S. H. Jin S. M. Suh S. Bae J. C. Park C. G. (2013). Co-transplantation of bone marrow-derived endothelial progenitor cells improves revascularization and organization in islet grafts. Am. J. Transpl. 13 (6), 1429–1440. 10.1111/ajt.12222 23601171

[B107] Okajima Y. Matsuzaka T. Miyazaki S. Motomura K. Ohno H. Sharma R. (2022). Morphological and functional adaptation of pancreatic islet blood vessels to insulin resistance is impaired in diabetic db/db mice. Biochim. Biophys. Acta Mol. Basis Dis. 1868 (4), 166339. 10.1016/j.bbadis.2022.166339 35017029

[B108] Olerud J. Mokhtari D. Johansson M. Christoffersson G. Lawler J. Welsh N. (2011). Thrombospondin-1: an islet endothelial cell signal of importance for beta-cell function. Diabetes 60 (7), 1946–1954. 10.2337/db10-0277 21617177 PMC3121439

[B109] Paget M. B. Murray H. E. Bailey C. J. Downing R. (2022). From insulin injections to islet transplantation: an overview of the journey. Diabetes Obes. Metab. 24 (Suppl. 1), 5–16. 10.1111/dom.14526 34431589

[B110] Pagliuca F. W. Millman J. R. Gürtler M. Segel M. Van Dervort A. Ryu J. H. (2014). Generation of functional human pancreatic beta cells *in vitro* . Cell 159 (2), 428–439. 10.1016/j.cell.2014.09.040 25303535 PMC4617632

[B111] Pan F. C. Wright C. (2011). Pancreas organogenesis: from bud to plexus to gland. Dev. Dyn. 240 (3), 530–565. 10.1002/dvdy.22584 21337462

[B112] Penko D. Mohanasundaram D. Sen S. Drogemuller C. Mee C. Bonder C. S. (2011). Incorporation of endothelial progenitor cells into mosaic pseudoislets. Islets 3 (3), 73–79. 10.4161/isl.3.3.15392 21478677

[B113] Penko D. Rojas-Canales D. Mohanasundaram D. Peiris H. S. Sun W. Y. Drogemuller C. J. (2015). Endothelial progenitor cells enhance islet engraftment, influence beta-cell function, and modulate islet connexin 36 expression. Cell Transpl. 24 (1), 37–48. 10.3727/096368913X673423 24069942

[B114] Pepper A. R. Bruni A. Shapiro A. M. J. (2018). Clinical islet transplantation: is the future finally now? Curr. Opin. Organ Transpl. 23 (4), 428–439. 10.1097/MOT.0000000000000546 29847441

[B115] Pepper A. R. Gala-Lopez B. Pawlick R. Merani S. Kin T. Shapiro A. M. J. (2015). A prevascularized subcutaneous device-less site for islet and cellular transplantation. Nat. Biotechnol. 33 (5), 518–523. 10.1038/nbt.3211 25893782

[B116] Phelps E. A. Headen D. M. Taylor W. R. Thulé P. M. García A. J. (2013). Vasculogenic bio-synthetic hydrogel for enhancement of pancreatic islet engraftment and function in type 1 diabetes. Biomaterials 34 (19), 4602–4611. 10.1016/j.biomaterials.2013.03.012 23541111 PMC3628538

[B117] Phelps E. A. Templeman K. L. Thulé P. M. García A. J. (2015). Engineered VEGF-releasing PEG-MAL hydrogel for pancreatic islet vascularization. Drug Deliv. Transl. Res. 5 (2), 125–136. 10.1007/s13346-013-0142-2 25787738 PMC4366610

[B118] Pileggi A. Molano R. D. Ricordi C. Zahr E. Collins J. Valdes R. (2006). Reversal of diabetes by pancreatic islet transplantation into a subcutaneous, neovascularized device. Transplantation 81 (9), 1318–1324. 10.1097/01.tp.0000203858.41105.88 16699461

[B119] Porter J. M. Yitayew M. Tabrizian M. (2023). Renewable human cell model for type 1 diabetes research: EndoC-βh5/HUVEC coculture spheroids. J. Diabetes Res. 2023, 6610007. 10.1155/2023/6610007 38162632 PMC10757655

[B120] Powers A. C. (2021). Type 1 diabetes mellitus: much progress, many opportunities. J. Clin. Invest 131 (8), e142242. 10.1172/JCI142242 33759815 PMC8262558

[B121] Powers A. C. Brissova M. (2021). Microvessels enhance vascularization and function of transplanted insulin-producing cells. Cell Metab. 33 (11), 2103–2105. 10.1016/j.cmet.2021.10.013 34731653

[B122] Quaranta P. Antonini S. Spiga S. Mazzanti B. Curcio M. Mulas G. (2014). Co-transplantation of endothelial progenitor cells and pancreatic islets to induce long-lasting normoglycemia in streptozotocin-treated diabetic rats. PLoS One 9 (4), e94783. 10.1371/journal.pone.0094783 24733186 PMC3986409

[B123] Quintard C. Tubbs E. Jonsson G. Jiao J. Wang J. Werschler N. (2024). A microfluidic platform integrating functional vascularized organoids-on-chip. Nat. Commun. 15 (1), 1452. 10.1038/s41467-024-45710-4 38365780 PMC10873332

[B124] Rambol M. H. Han E. Niklason L. E. (2020). Microvessel network formation and interactions with pancreatic islets in three-dimensional chip cultures. Tissue Eng. Part A 26 (9-10), 556–568. 10.1089/ten.TEA.2019.0186 31724494 PMC7249478

[B125] Rankin M. M. Kushner J. A. (2009). Adaptive beta-cell proliferation is severely restricted with advanced age. Diabetes 58 (6), 1365–1372. 10.2337/db08-1198 19265026 PMC2682671

[B126] Rezania A. Bruin J. E. Arora P. Rubin A. Batushansky I. Asadi A. (2014). Reversal of diabetes with insulin-producing cells derived *in vitro* from human pluripotent stem cells. Nat. Biotechnol. 32 (11), 1121–1133. 10.1038/nbt.3033 25211370

[B127] Rickels M. R. Robertson R. P. (2019). Pancreatic islet transplantation in humans: recent progress and future directions. Endocr. Rev. 40 (2), 631–668. 10.1210/er.2018-00154 30541144 PMC6424003

[B128] Riley K. G. Pasek R. C. Maulis M. F. Peek J. Thorel F. Brigstock D. R. (2015). Connective tissue growth factor modulates adult beta-cell maturity and proliferation to promote beta-cell regeneration in mice. Diabetes 64 (4), 1284–1298. 10.2337/db14-1195 25392241 PMC4375083

[B129] Sakhneny L. Epshtein A. Landsman L. (2021). Pericytes contribute to the islet basement membranes to promote beta-cell gene expression. Sci. Rep. 11 (1), 2378. 10.1038/s41598-021-81774-8 33504882 PMC7840750

[B130] Salamone M. Rigogliuso S. Nicosia A. Campora S. Bruno C. M. Ghersi G. (2021). 3D collagen hydrogel promotes *in vitro* langerhans islets vascularization through ad-MVFs angiogenic activity. Biomedicines 9 (7), 739. 10.3390/biomedicines9070739 34199087 PMC8301445

[B131] Seo H. Son J. Park J. K. (2020). Controlled 3D co-culture of beta cells and endothelial cells in a micropatterned collagen sheet for reproducible construction of an improved pancreatic pseudo-tissue. Apl. Bioeng. 4 (4), 046103. 10.1063/5.0023873 33195961 PMC7647615

[B132] Shapiro A. M. Lakey J. R. Ryan E. A. Korbutt G. S. Toth E. Warnock G. L. (2000). Islet transplantation in seven patients with type 1 diabetes mellitus using a glucocorticoid-free immunosuppressive regimen. N. Engl. J. Med. 343 (4), 230–238. 10.1056/NEJM200007273430401 10911004

[B133] Shapiro A. M. Pokrywczynska M. Ricordi C. (2017). Clinical pancreatic islet transplantation. Nat. Rev. Endocrinol. 13 (5), 268–277. 10.1038/nrendo.2016.178 27834384

[B134] Shepherd B. R. Chen H. Y. S. Smith C. M. Gruionu G. Williams S. K. Hoying J. B. (2004). Rapid perfusion and network remodeling in a microvascular construct after implantation. Arterioscler. Thromb. Vasc. Biol. 24 (5), 898–904. 10.1161/01.ATV.0000124103.86943.1e 14988090

[B135] Smink A. M. Li S. Hertsig D. T. de Haan B. J. Schwab L. van Apeldoorn A. A. (2017). The efficacy of a prevascularized, retrievable poly(D,L,-lactide-co-epsilon-caprolactone) subcutaneous scaffold as transplantation site for pancreatic islets. Transplantation 101 (4), e112–e119. 10.1097/TP.0000000000001663 28207637 PMC7228571

[B136] Soltanian A. Ghezelayagh Z. Mazidi Z. Halvaei M. Mardpour S. Ashtiani M. K. (2019). Generation of functional human pancreatic organoids by transplants of embryonic stem cell derivatives in a 3D-printed tissue trapper. J. Cell Physiol. 234 (6), 9564–9576. 10.1002/jcp.27644 30362564

[B137] Song H. J. Xue W. J. Li Y. Tian X. H. Song Y. Ding X. M. (2009). Improved islet survival and funtion with rat endothelial cells *in vitro* co-culture. Transpl. Proc. 41 (10), 4302–4306. 10.1016/j.transproceed.2009.09.071 20005388

[B138] Song W. Chiu A. Wang L. H. Schwartz R. E. Li B. Bouklas N. (2019). Engineering transferrable microvascular meshes for subcutaneous islet transplantation. Nat. Commun. 10 (1), 4602. 10.1038/s41467-019-12373-5 31601796 PMC6787187

[B139] Sorenby A. K. Kumagai-Braesch M. Sharma A. Hultenby K. R. Wernerson A. M. Tibell A. B. (2008). Preimplantation of an immunoprotective device can lower the curative dose of islets to that of free islet transplantation: studies in a rodent model. Transplantation 86 (2), 364–366. 10.1097/TP.0b013e31817efc78 18645504

[B140] Sorenson R. L. Brelje T. C. (1997). Adaptation of islets of Langerhans to pregnancy: beta-cell growth, enhanced insulin secretion and the role of lactogenic hormones. Horm. Metab. Res. 29 (6), 301–307. 10.1055/s-2007-979040 9230352

[B141] Spelios M. G. (2018). Human EndoC-betaH1 beta-cells form pseudoislets with improved glucose sensitivity and enhanced GLP-1 signaling in the presence of islet-derived endothelial cells. Am. J. Physiol. Endocrinol. Metab. 314 (5), E512–E521. 10.1152/ajpendo.00272.2017 29351476

[B142] Su D. Zhang N. He J. Qu S. Slusher S. Bottino R. (2007). Angiopoietin-1 production in islets improves islet engraftment and protects islets from cytokine-induced apoptosis. Diabetes 56 (9), 2274–2283. 10.2337/db07-0371 17596403

[B143] Sun X. Aghazadeh Y. Nunes S. S. (2022). Isolation of ready-made rat microvessels and its applications in effective *in vivo* vascularization and in angiogenic studies *in vitro* . Nat. Protoc. 17 (12), 2721–2738. 10.1038/s41596-022-00743-1 36224469

[B144] Sun X. Wu J. Qiang B. Romagnuolo R. Gagliardi M. Keller G. (2020). Transplanted microvessels improve pluripotent stem cell-derived cardiomyocyte engraftment and cardiac function after infarction in rats. Sci. Transl. Med. 12 (562), eaax2992. 10.1126/scitranslmed.aax2992 32967972

[B145] Takahashi Y. Sekine K. Kin T. Takebe T. Taniguchi H. (2018b). Self-condensation culture enables vascularization of tissue fragments for efficient therapeutic transplantation. Cell Rep. 23 (6), 1620–1629. 10.1016/j.celrep.2018.03.123 29742420 PMC8289710

[B146] Takahashi Y. Takebe T. Taniguchi H. (2018a). Methods for generating vascularized islet-like organoids via self-condensation. Curr. Protoc. Stem Cell Biol. 45 (1), e49. 10.1002/cpsc.49 30040240

[B147] Takebe T. Enomura M. Yoshizawa E. Kimura M. Koike H. Ueno Y. (2015). Vascularized and complex organ buds from diverse tissues via mesenchymal cell-driven condensation. Cell Stem Cell 16 (5), 556–565. 10.1016/j.stem.2015.03.004 25891906

[B148] Takebe T. Zhang R. R. Koike H. Kimura M. Yoshizawa E. Enomura M. (2014). Generation of a vascularized and functional human liver from an iPSC-derived organ bud transplant. Nat. Protoc. 9 (2), 396–409. 10.1038/nprot.2014.020 24457331

[B149] Talavera-Adame D. Woolcott O. O. Ignatius-Irudayam J. Arumugaswami V. Geller D. H. Dafoe D. C. (2016). Effective endothelial cell and human pluripotent stem cell interactions generate functional insulin-producing beta cells. Diabetologia 59 (11), 2378–2386. 10.1007/s00125-016-4078-1 27567623 PMC5506104

[B150] Talavera-Adame D. Wu G. He Y. Ng T. T. Gupta A. Kurtovic S. (2011). Endothelial cells in co-culture enhance embryonic stem cell differentiation to pancreatic progenitors and insulin-producing cells through BMP signaling. Stem Cell Rev. Rep. 7 (3), 532–543. 10.1007/s12015-011-9232-z 21298405 PMC3137775

[B151] Tao T. Deng P. Wang Y. Zhang X. Guo Y. Chen W. (2022). Microengineered multi-organoid system from hiPSCs to recapitulate human liver-islet Axis in normal and type 2 diabetes. Adv. Sci. (Weinh) 9 (5), e2103495. 10.1002/advs.202103495 34951149 PMC8844474

[B152] Tao T. Wang Y. Chen W. Li Z. Su W. Guo Y. (2019). Engineering human islet organoids from iPSCs using an organ-on-chip platform. Lab. Chip 19 (6), 948–958. 10.1039/c8lc01298a 30719525

[B153] Tschen S. I. Dhawan S. Gurlo T. Bhushan A. (2009). Age-dependent decline in beta-cell proliferation restricts the capacity of beta-cell regeneration in mice. Diabetes 58 (6), 1312–1320. 10.2337/db08-1651 19228811 PMC2682690

[B154] Urbanczyk M. Abuhelou A. Köninger M. Jeyagaran A. Carvajal-Berrio D. Kim E. (2024). Heterogeneity of endothelial cells impacts the functionality of human pancreatic *in vitro* models. Tissue Eng. Part A. 10.1089/ten.tea.2024.0176 39453887

[B155] Urbanczyk M. Zbinden A. Layland S. L. Duffy G. Schenke-Layland K. (2020). Controlled heterotypic pseudo-islet assembly of human β-cells and human umbilical vein endothelial cells using magnetic levitation. Tissue Eng. Part A 26 (7-8), 387–399. 10.1089/ten.TEA.2019.0158 31680653 PMC7187983

[B156] Vartanian K. B. Kirkpatrick S. J. McCarty O. J. T. Vu T. Q. Hanson S. R. Hinds M. T. (2009). Distinct extracellular matrix microenvironments of progenitor and carotid endothelial cells. J. Biomed. Mater Res. A 91 (2), 528–539. 10.1002/jbm.a.32225 18985765

[B157] Vasavada R. C. Garcia-Ocaña A. Zawalich W. S. Sorenson R. L. Dann P. Syed M. (2000). Targeted expression of placental lactogen in the beta cells of transgenic mice results in beta cell proliferation, islet mass augmentation, and hypoglycemia. J. Biol. Chem. 275 (20), 15399–15406. 10.1074/jbc.275.20.15399 10809775

[B158] Vasir B. Jonas J. C. Steil G. M. Hollister-Lock J. Hasenkamp W. Sharma A. (2001). Gene expression of VEGF and its receptors Flk-1/KDR and Flt-1 in cultured and transplanted rat islets. Transplantation 71 (7), 924–935. 10.1097/00007890-200104150-00018 11349728

[B159] Vertex (2024). Vertex launches pivotal trial for stem-cell derived islet therapy.

[B160] Vlahos A. E. Cober N. Sefton M. V. (2017). Modular tissue engineering for the vascularization of subcutaneously transplanted pancreatic islets. Proc. Natl. Acad. Sci. U. S. A. 114 (35), 9337–9342. 10.1073/pnas.1619216114 28814629 PMC5584405

[B161] Wang B. Song X. Zhang X. Li Y. Xu M. Liu X. (2024a). Harnessing the benefits of glycine supplementation for improved pancreatic microcirculation in type 1 diabetes mellitus. Microvasc. Res. 151, 104617. 10.1016/j.mvr.2023.104617 37918522

[B162] Wang D. Wang J. Bai L. Pan H. Feng H. Clevers H. (2020). Long-term expansion of pancreatic islet organoids from resident Procr(+) progenitors. Cell 180 (6), 1198–1211. 10.1016/j.cell.2020.02.048 32200801

[B163] Wang J. Wang D. Chen X. Yuan S. Bai L. Liu C. (2022). Isolation of mouse pancreatic islet Procr(+) progenitors and long-term expansion of islet organoids *in vitro* . Nat. Protoc. 17 (5), 1359–1384. 10.1038/s41596-022-00683-w 35396545

[B164] Wang L. Wan J. Xu Y. Huang Y. Wang D. Zhu D. (2024c). Endothelial cells promote pseudo-islet function through BTC-EGFR-JAK/STAT signaling pathways. Ann. Biomed. Eng. 52 (9), 2610–2626. 10.1007/s10439-024-03548-3 38829457

[B165] Wang L. H. Marfil-Garza B. A. Ernst A. U. Pawlick R. L. Pepper A. R. Okada K. (2023). Inflammation-induced subcutaneous neovascularization for the long-term survival of encapsulated islets without immunosuppression. Nat. Biomed. Eng. 8, 1266–1284. 10.1038/s41551-023-01145-8 38052996

[B166] Wang Q. Huang Y. X. Liu L. Zhao X. H. Sun Y. Mao X. (2024b). Pancreatic islet transplantation: current advances and challenges. Front. Immunol. 15, 1391504. 10.3389/fimmu.2024.1391504 38887292 PMC11180903

[B167] Wang S. Du Y. Zhang B. Meng G. Liu Z. Liew S. Y. (2024d). Transplantation of chemically induced pluripotent stem-cell-derived islets under abdominal anterior rectus sheath in a type 1 diabetes patient. Cell 187 (22), 6152–6164 e18. 10.1016/j.cell.2024.09.004 39326417

[B168] Wassmer C. H. Lebreton F. Bellofatto K. Perez L. Cottet-Dumoulin D. Andres A. (2021). Bio-engineering of pre-vascularized islet organoids for the treatment of type 1 diabetes. Transpl. Int. 35, 10214. 10.3389/ti.2021.10214 35185372 PMC8842259

[B169] Weaver J. D. Headen D. M. Aquart J. Johnson C. T. Shea L. D. Shirwan H. (2017). Vasculogenic hydrogel enhances islet survival, engraftment, and function in leading extrahepatic sites. Sci. Adv. 3 (6), e1700184. 10.1126/sciadv.1700184 28630926 PMC5457148

[B170] Weaver J. D. Headen D. M. Hunckler M. D. Coronel M. M. Stabler C. L. García A. J. (2018). Design of a vascularized synthetic poly(ethylene glycol) macroencapsulation device for islet transplantation. Biomaterials 172, 54–65. 10.1016/j.biomaterials.2018.04.047 29715595 PMC5967258

[B171] Wieland F. C. Sthijns M. M. J. P. E. Geuens T. van Blitterswijk C. A. LaPointe V. L. S. (2021). The role of pancreatic alpha cells and endothelial cells in the reduction of oxidative stress in pseudoislets. Front. Bioeng. Biotechnol. 9, 729057. 10.3389/fbioe.2021.729057 34568302 PMC8458707

[B172] Wrublewsky S. Weinzierl A. Hornung I. Prates-Roma L. Menger M. D. Laschke M. W. (2022). Co-transplantation of pancreatic islets and microvascular fragments effectively restores normoglycemia in diabetic mice. NPJ Regen. Med. 7 (1), 67. 10.1038/s41536-022-00262-3 36333332 PMC9636251

[B173] Xiong Y. Scerbo M. J. Seelig A. Volta F. O'Brien N. Dicker A. (2020). Islet vascularization is regulated by primary endothelial cilia via VEGF-A-dependent signaling. Elife 9, e56914. 10.7554/eLife.56914 33200981 PMC7695455

[B174] Yancopoulos G. D. Davis S. Gale N. W. Rudge J. S. Wiegand S. J. Holash J. (2000). Vascular-specific growth factors and blood vessel formation. Nature 407 (6801), 242–248. 10.1038/35025215 11001067

[B175] Yang L. Han Y. Zhang T. Dong X. Ge J. Roy A. (2024). Human vascularized macrophage-islet organoids to model immune-mediated pancreatic β cell pyroptosis upon viral infection. Cell Stem Cell 31, 1612–1629.e8. 10.1016/j.stem.2024.08.007 39232561 PMC11546835

[B176] Yoshihara E. O'Connor C. Gasser E. Wei Z. Oh T. G. Tseng T. W. (2020). Immune-evasive human islet-like organoids ameliorate diabetes. Nature 586 (7830), 606–611. 10.1038/s41586-020-2631-z 32814902 PMC7872080

[B177] Yoshitomi H. Zaret K. S. (2004). Endothelial cell interactions initiate dorsal pancreas development by selectively inducing the transcription factor Ptf1a. Development 131 (4), 807–817. 10.1242/dev.00960 14736742

[B178] Yu X. X. Xu C. R. (2020). Understanding generation and regeneration of pancreatic beta cells from a single-cell perspective. Development 147 (7), dev179051. 10.1242/dev.179051 32280064

[B179] Zbinden A. Urbanczyk M. Layland S. L. Becker L. Marzi J. Bosch M. (2021). Collagen and endothelial cell coculture improves β-cell functionality and rescues pancreatic extracellular matrix. Tissue Eng. Part A 27 (13-14), 977–991. 10.1089/ten.TEA.2020.0250 33023407

[B180] Zhang N. Richter A. Suriawinata J. Harbaran S. Altomonte J. Cong L. (2004). Elevated vascular endothelial growth factor production in islets improves islet graft vascularization. Diabetes 53 (4), 963–970. 10.2337/diabetes.53.4.963 15047611

[B181] Zhou Q. Brown J. Kanarek A. Rajagopal J. Melton D. A. (2008). *In vivo* reprogramming of adult pancreatic exocrine cells to beta-cells. Nature 455 (7213), 627–632. 10.1038/nature07314 18754011 PMC9011918

[B182] Zou W. Liu B. Wang Y. Shi F. Pang S. (2022). Metformin attenuates high glucose-induced injury in islet microvascular endothelial cells. Bioengineered 13 (2), 4385–4396. 10.1080/21655979.2022.2033411 35139776 PMC8973819

