# Supplementary material for: The role of endothelial cells in pancreatic islet development, transplantation and culture
Source: Front Cell Dev Biol. 2025 Apr 22;13:1558137. doi: 10.3389/fcell.2025.1558137 (PMC12052768; doi:10.3389/fcell.2025.1558137)
Supplement: Supplementary file 1 [file Table1.docx]

**Supplementary Table 1: Summary of Vascular Engineering for Islet Transplantation**

| **Reference** | **The source and isolation of the EC** | **The source of the b-cell** | **Details of the Engineering** | **Function of the**  **engineered EC**  **(characterization/period observation)** | **Organoid/islet function**  **after TP** | **Contributions**  **(Advantages-A, Limitations-L, Scalability-S)** |
| --- | --- | --- | --- | --- | --- | --- |
| **2.3.1 Utilizing Biomaterials to Promote Subcutaneous Neovascularization** | | | | | | |
| Mahou et al; 2017 [119] | - Mice - S.c. autogenous EC - Primary | Mice islets | - A semi-interpenetrating polymer network (SIPN) - S.c. site(co-TP) - Diabetic mice (STZ) | - Vessel number ↑ - Vessel density ↑ - Formation of a perfusable vasculature ↑ - EC marker: CD31/GSL-1 staining - 7/14/21 days after TP | - Islet viability ↑ - Insulin stimulation - - A progressive return to normoglycemia - Reversal of diabetes (10 days) | A: SIPN can promote s.c. vascularization and is feasible for islet TP  A: A novel vascular regenerating delivery vehicle was prepared and characterized  L: The practicality of SIPN in longer durations and various diabetic models has not been demonstrated  S: Not strong |
| Kawakami et al; 2000/2001 [120, 121] | - Rat - S.c. autogenous EC - Primary | Rat islets | - A bFGF-releasing device to induce neovascularization (enclosing bFGF in a polyethylene terephthalate mesh bag coated with polyvinylalcohol hydrogel) - S.c. site (1 week before TP) - Diabetic rats (STZ) | - A thick, well-vascularized capsule was observed - EC marker: H&E staining - 7 days after TP | - Restoration of normoglycemia - Body weight ↑ - Reversal of diabetes (42/100 days) | A: A bFGF-releasing device can be formed well-vascularized capsule  A: Establishment of this new method for successful s.c. islet transplantation  L: The long-term function of islets is under further investigation.  L: The change of the microenvironment is unclear.  S: Not strong |
| Luan et al; 2014 [122] | - Rat - S.c. autogenous EC - Primary | Rat islets | - Two agarose rods with bFGF and heparin to induce Neovascularization - S.c. site(1 week before TP) - Diabetic rats (STZ) | - Blood capillaries ↑ - Functional vasculature ↑ - Engraftment and revascularization of allogeneic islets ↑ - EC marker: Lectin staining - 7/30 days after TP | - Restoration of normoglycemia - GTT ↑ - Insulin levels - - Reversal of diabetes (200 days) | A: Well-vascularized pockets by implantation of agarose rods containing bFGF and heparin  A: First report to achieve long-term allogeneic islet graft survival without immunosuppressive treatment  L: The mechanism remains to be elucidated.  L: Vascular structures damage and inflammation during device removal.  S: Not strong |
| **2.3.2 Utilizing Bio-devices to Promote Subcutaneous Neovascularization** | | | | | | |
| Craig et al; 2005 [123] | - Rat - S.c. autogenous EC - Primary | Rat islets | - A cylindrical stainless steel with islet - S.c. site (60 days before TP) - Diabetic rat (STZ) | - EC marker: H&E staining - 7 days after TP | - Restoration of normoglycemia - Islet function ↑ - GTT ↑ - Reversal of diabetes (60 days) | A: β cell function in this device compared favorably with the function of islets transplanted to the renal subscapular space  L: Vascular structures damage and inflammation during device removal.  S: Not strong |
| Pileggi et al; 2006 [124] | - Rat - S.c. autogenous EC - Primary | Rat islets | - A cylindrical stainless-steel mesh induce neovascularization - S.c. site (40 days before TP) - Diabetic rats (STZ) | - Connective tissue and neovascularization embedded the device - EC marker: Macroscopic appearance/vWF staining - 80/170 days after TP | - Restoration of normoglycemia - Maintained long-term functionality - Body weight ↑ - GTT ↑ - Reversal of diabetes (>160 days) | A: Connective tissue and neovascularization embedded the device.  A: Establishment of this new method for successful s.c. islet transplantation.  L: The mechanism remains to be elucidated.  S: Not strong |
| Sorenby et al; 2008 [126] | - Rat - S.c. autogenous EC - Primary | Rat islets | - TheraCyte immunoprotective devices - S.c. site (40 days before TP) - Diabetic rats (STZ) | - Neo-vascularizing membrane - EC marker: Histological sections - 28 days after TP | - The dose of islets reduced 10 times - Reversal of diabetes (30 days) | A: The dose of islets required for TheraCyte devices is significantly reduced.  A; The encapsulated islets were reduced 10 times, and equaled to kidney capsule.  L: The long-term effects of the device were not observed.  S: Not strong |
| Andrew et al; 2015 [125] | - Mouse - S.c. autogenous EC - Primary | Mice islets | - A subcutaneous cell pouch device - S.c. site (3-4 weeks before TP) - Diabetic mice (STZ) | - Blood vessels ↑ - Previsualize of Cell Pouch - EC marker: vMF staining - 35/100 days after TP | - Restoration of normoglycemia (100 days) - GTT ↑ - Comparable to renal subcapsular islet grafts | A: Cell Pouch forms a suitable environment for islet engraftment  A: A potential alternative to the intraportal site for islet and future stem cell therapies  L: The delay in achieving insulin independence after islet transplantation into the CP.  S: Not strong |
| Smink et al; 2017 [127] | - Mouse - S.c. autogenous EC - Primary | Mice islets | - Poly (D, L-lactide-co-ε-caprolactone) (PDLLCL) scaffold (PE rods with high hydrophobicity) - S.c. site (4 weeks before TP) - Diabetic mice (STZ) | - Vascularization ↑ - EC marker: Macroscopic appearance - 1 month after TP | - Restoration of normoglycemia (70 days) - GTT ↑ | A: (PDLLCL) scaffold maintains viability and function of islets in the subcutaneous site.  L: The subcutaneous scaffold was less effective as a transplantation site compared to the kidney capsule.  S: Not strong |
| Liu et al; 2020 [128] | - Mice - S.c. autogenous EC - Primary | Mouse islets | - A prevascularized tissue-engineered chamber (TEC) (filled with Growth Factor-Matrigel™) - S.c. site (28 days before TP) - Diabetic mice (STZ) | - Microvascularized network established - VEGF ↑ - CD31 ↑ - EC marker: CD31/VEFG staining - 7/14/28/35 days after TP | - Restoration of normoglycemia (90 days) - GTT ↑ - Long-term survival and functional of allografts and xenografts - Immune tolerance ↑ | A: TEC is a suitable site for transplantation  A: TECs induced long-term recipient-specific immune tolerance L: The autoimmune diabetes models have not yet been studied. S: The uncertainty of the location and size of the chamber，but can be multiple transplantation. |
| Pepper et al; 2015 [129]  Wang et al; 2023 [131] | - Mice - S.c. autogenous EC - Primary | Encapsulated and human islets | - A prevascularized s.c. device (a hollow nylon catheter); SHEATH system - S.c. site (4-6 weeks before TP) - Diabetic mice (STZ) | - A vascularized pocket - Vessel number ↑ - Local O2 ↑ - EC marker: CD31 staining - 86/127 days after TP | - Reversal of diabetes (200 days) - Long-term islet survival - GTT ↑ - Immunosuppression ↑ - Can be replaced | A: A new method for pre-vascularization of s.c. transplants.  A: Clinical translation of s.c. islet transplantation without immunosuppression.  A: The SHEATH system can be replaced.  L: Experiments were conducted solely in large animal models, which is insufficient for clinical translation.  L: The device design should be modified to accommodate a clinically curative cell load.  S: Have the scalability using a large animal (minipig) model.  S: Have launched clinical trial (NCT05073302) |
| Song et al；2019 [132] | - Human - HUVEC - iPSC-EC [182] - Primary | Rat islets | - Microvascular mesh was established *in vitro* - S.c. site - Diabetic mice (STZ) | - Angiogenesis and vascularization ↑ - Anastomoses with host vasculature ↑ - EC marker: CD31 staining - 42/91/112 days after TP | - Restoration of normoglycemia (90 days) - Blood glucose ↑ - GTT ↑ | A: Construction of functional microvascular mesh *in vitro.*  A: The microvascular mesh enhanced the function of s.c. islet transplantation.  L: There is an issue of immune rejection.  L: Technological complexity and scalability.  S: Can be fabricated in larger sizes, but only using iPSC-EC.  S: Can expanded to other bioengineering fields. |

Endothelial cells (ECs); Human umbilical vein endothelial cells (HUVEC); Subcutaneous (s.c.); Transplantation (TP); Streptozotocin (STZ); basic fibroblast growth factor (bFGF); Hematoxylin and eosin (H&E); Glucose tolerance test (GTT); von Willebrand factor (vWF); Induced pluripotent stem cells (iPSCs).
